# A Metabolomics Approach to Investigate Kukoamine B—A Potent Natural Product With Anti-diabetic Properties

**DOI:** 10.3389/fphar.2018.01575

**Published:** 2019-01-22

**Authors:** Yuan-Yuan Li, Delisha A. Stewart, Xiao-Min Ye, Li-Hua Yin, Wimal W. Pathmasiri, Susan L. McRitchie, Timothy R. Fennell, Hon-Yeung Cheung, Susan J. Sumner

**Affiliations:** ^1^NIH Eastern Regional Comprehensive Metabolomics Resource Core, Nutrition Research Institute, Department of Nutrition, University of North Carolina at Chapel Hill, Kannapolis, NC, United States; ^2^Department of Pharmacology, Wuhan Institute for Drug and Medical Device Control, Wuhan, China; ^3^Analytical Chemistry and Pharmaceutics, RTI International, Research Triangle Park, Durham, NC, United States; ^4^Department of Biomedical Science, City University of Hong Kong, Kowloon, Hong Kong

**Keywords:** kukoamine B, type 2 diabetes mellitus, db/db mouse, metabolomics, lipidomics, cytokine array

## Abstract

Due to the surge in type 2 diabetes mellitus (T2DM), treatments for chronic metabolic dysregulations with fewer side-effects are sought. Lycii Cortex (LyC), a traditional Chinese Medicine (TCM) herb has a long history of being widely prescribed to treat T2DM as alternative medicine; however, the bioactive molecules and working mechanism remained unknown. Previous studies revealed kukoamine B (KB) as a major and featured compound for LyC with bioactivities for anti-oxidation and acute inflammation, which may be related to anti-diabetes properties. This study aims to understand the efficacy and the mode of action of KB in the diabetic (db/db) mouse model using a metabolomics approach. Parallel comparison was conducted using the first-line anti-diabetic drugs, metformin and rosligtazone, as positive controls. The db/db mice were treated with KB (50 mg kg^−1^ day^−1^) for 9 weeks. Bodyweight and fasting blood glucose were monitored every 5 and 7 days, respectively. Metabolomics and high-throughput molecular approaches, including lipidomics, targeted metabolomics (Biocrates p180), and cytokine profiling were applied to measure the alteration of serum metabolites and inflammatory biomarkers between different treatments vs. control (db/db mice treated with vehicle). After 9 weeks of treatment, KB lowered blood glucose, without the adverse effects of bodyweight gain and hepatomegaly shown after rosiglitazone treatment. Lipidomics analysis revealed that KB reduced levels of circulating triglycerides, cholesterol, phosphatidylethanolamine, and increased levels of phosphatidylcholines. KB also increased acylcarnitines, and reduced systemic inflammation (cytokine array). Pathway analysis suggested that KB may regulate nuclear transcription factors (e.g., NF-κB and/or PPAR) to reduce inflammation and facilitate a shift toward metabolic and inflammatory homeostasis. Comparison of KB with first-line drugs suggests that rosiglitazone may over-regulate lipid metabolism and anti-inflammatory responses, which may be associated with adverse side effects, while metformin had less impact on lipid and anti-inflammation profiles. Our research from holistic and systemic views supports the conclusion that KB is the bioactive compound of LyC for managing T2DM, and suggests KB as a nutraceutical or a pharmaceutical candidate for T2D treatment. In addition, our research provides insights related to metformin and rosiglitazone action, beyond lowering blood glucose.

## Introduction

Systemic metabolic diseases, especially type 2 diabetes mellitus (T2DM), have become a global public health issue. According to the International Diabetes Federation, there are approximately 415 million diabetic patients in the world, and the number is expected to rise to 642 million by 2,040 (Forces, 2015).

T2DM presents a feature of uncontrolled high blood sugar accompanied with long-term complications such as cardiovascular diseases, diabetic retinopathy, and diabetic nephropathy. Although the pathogenesis is not yet fully understood, obesity, insulin resistance, metabolic disorder, and chronic low-grade inflammation are associated with T2DM (Esser et al., 2014). One of the most recognized theories is lipotoxicity (Schooneman et al., 2012; McArdle et al., 2013). Here, the over-accumulation of lipids and lipid metabolites interrupts normal cell signal transitions and stimulates immune cell infiltration which produces chronic inflammation that further exacerbates metabolic dysfunction (McArdle et al., 2013). In this regard, correcting lipid dysregulation and reducing inflammation are keys for managing T2DM, especially in early stages.

In addition to changing diet and lifestyle, dietary supplements and drug treatments provide a complementary strategy (Balducci et al., 2010; Poulsen et al., 2013). Most prescribed anti-diabetic drugs work on glycemic control through inhibiting intestinal adsorption (acarbose) (Bischoff, 1995), stimulating pancreatic insulin secretion (sitagliptin and glipizide) (Thulé and Umpierrez, 2014), sensitizing insulin receptors (metformin and rosiglitazone) (Ahmadian et al., 2013), and inhibiting liver gluconeogenesis (metformin) (Hundal et al., 2000). However, adverse effects, including gastrointestinal reactions, bodyweight gain, blood glucose fluctuation (Bischoff, 1995; Thulé and Umpierrez, 2014), and risks in cardiovascular disease (Gerstein et al., 2008; Ahmadian et al., 2013) have limited their use for long-term treatment of T2DM.

As an alternative approach, natural supplements originating from traditional medicine or fruits or vegetables have been widely used to maintain T2DM (Yeh et al., 2003; Yin et al., 2008; Ota and Ulrih, 2017), because of reduced cost and easier accessibility compared to prescribed medications. In fact, many pharmaceuticals commonly used today are structurally derived from natural molecules, such as metformin. Some natural products, such as flavonoids, polyphenols, and organic acids, have shown *in vitro* efficacy involving actions of reducing glucose absorption (Zhang et al., 2016), alleviating insulin resistance (Zhang et al., 2012), oxidative stress (Ramful et al., 2010; Li et al., 2015), and inflammation (Zagotta et al., 2015). However, in-depth and holistic *in vivo* studies are still required to validate the efficacies and safety and to understand the modes of action, because T2DM is a systemic metabolic disorder. Such investigations are extremely important before such natural products are recommended for clinical trial or for usage in treatment and/or prevention or attenuation of diabetes.

In recent years, the rise of metabolomics, a high throughput technology for measuring all or phenotype-relevant metabolites at one time (Bain et al., 2009; Newgard, 2017), has provided novel tools to understand the action of drug or nutritional intervention by characterizing the perturbed metabolites between groups with and without treatment (Kaddurah-Daouk and Weinshilboum, 2014, 2015). These perturbed metabolites can be further used to identify targets and pathways involved in efficacy, side reactions, and even toxicity (Nicholson et al., 2002; Stewart et al., 2015). The application of metabolomics to pharmacological studies therefore provides more comprehensive and in-depth information at a molecular level than an approach only focusing on one or several phenotypic outcomes, such as body weight, blood glucose, and enzyme activity, which are normally used to judge the efficacy or adverse effects of the treatments. Metabolomics, integrated with pathway analysis, enables novel directions in understanding the modes of action for traditional medicine, especially for study designs based on *in vivo* models and human subjects. For example, Su et al. used a metabolomics approach to reveal that baicalein can alleviate heavy metal-induced liver and kidney damage through regulating energy metabolism, choline metabolism, amino acid metabolism, and gut flora (Su et al., 2017). Xie et al. using GC-MS and LC-MS based metabolomics approaches, discovered a 2-week intervention with Pu-erh tea significantly changed human urinary global metabolite profiles, where the increase of nicotinic acid in urine after pu-erh tea intake may be responsible for cholesterol reducing and lipid-lowering effects, and the depleted concentration of 3-chloroytrosine may be associated with lowering triglyceride (TG) and low-density lipoprotein (LDL) (Xie et al., 2012).

Lycii Cortex (LyC) is an herb frequently prescribed in TCM to treat chronic metabolic diseases, e.g., diabetes and hypertension (Li et al., 2004; Potterat, 2009; Su et al., 2013), yet the mechanism has never been revealed. Our previous study revealed that kukoamines, especially kukoamine B, are the major compounds of LyC, accounting for approximately 2% of the herbal dry mass (Li et al., 2014). Recent research from our group and other research groups has revealed that KB (or its isomer kukoamine A) has bioactivities in anti-oxidation, anti-inflammation, and anti-insulin resistance (Liu et al., 2011; Li et al., 2015, 2017), which are highly associated with diabetes; however, direct *in vivo* evidence showing the beneficial effect of KB on T2DM from the viewpoint of metabolomics has not yet been found.

In the current study we used multi-metabolomics approaches, including lipidomics and a targeted panel (Biocrates p180 kit), as well as a molecular assay to profile cytokines, to study the effect of KB on the diabetic db/db mouse model. The perturbed lipids, metabolites, and cytokines were analyzed to determine the enriched biochemical pathways. Furthermore, KB was compared with the first-line anti-T2DM drugs metformin and rosiglitazone regarding efficacy, side effects, and modes of action.

## Methods

### Animals and Treatment

A spontaneous type 2 diabetic animal model, the db/db mouse model, was used in this study to simulate the hyperglycemia and dyslipidemia symptoms in human T2DM (Cefalu, 2006; Adam et al., 2016). Male, 4-week old db/db mice (BKS.Cg-m+/+ Leprdb/J, *n* = 50) and age- and strain-matched wild-type (WT) mice (C57BLKS/J-m+/+ db, *n* = 10) were purchased from the Mode Animal Research Center of Nanjing University (Nanjing, China). All mice were kept in a specific pathogen free (SPF) condition in the laboratory animal center located in the Wuhan Institute of Drug and Medical Device Control (WIDMDC, Wuhan, China), under regulated environmental conditions with temperature at 25°C, humidity at 50%, and a 12:12-h light-dark cycle. All mice were fed standard chow with *ad libitum* access to food and water. All mice had one-week adaptive feeding before receiving treatments. KB (95%) was purified from Lycii Cortex extract according to the patent awarded to Cheung et al. (US9012687B2) (Cheung et al., 2015). According to the literature, db/db mice start to present diabetic symptoms, such as hyperglycemia and weight gain, in week 4–6 (Adam et al., 2016). In our study, at the end of week-5, the bodyweight and fasting blood glucose from tail vein were measured according to standard protocol (Ayala et al., 2010). We found that db/db mice demonstrated significant diabetic phenotypes, showing 2-fold higher bodyweight and 3-fold higher blood glucose than the WT mice. At this time point, all diabetic mice were distributed randomly into 4 groups (*n* = 10 per group) and received gavage with 50 mg/kg/d of KB, 5 mg/kg/d of metformin, 5 mg/kg/d of rosiglitazone, or the same volume of saline as vehicle (negative control). WT mice (*n* = 10) were only treated with vehicle. KB dosage levels were selected according to previously published work by Liu et al. (2011). We investigated two concentrations (20 and 50 mg/Kg/day) in the whole animal study, and the high-dose group was selected for metabolomics study because of the more significant effect in controlling blood glucose. With the nutraceutical effect being our desired primary endpoint, the selection of dose-range for metformin and rosiglitazone were based on a small pilot study before doing the full animal study. We investigated two doses of 5 and 20 mg/Kg/day for metformin and rosiglitazone, respectively, and found the lower doses for both drugs demonstrated significant effects in lowering blood glucose compared to the untreated controls. The bodyweight of mice was measured every 5 days and the fasting blood glucose was measured weekly from tail vein using a glucose meter (Ayala et al., 2010). At the end of the experiment (9 weeks of treatment, mice age = 15 weeks), mice were fasted overnight and then euthanized by CO_2_ inhalation. Serum and tissues were collected, weighed, and snap-frozen at −80°C until analysis. Animal handling was conducted according to the “Guide for the Care and Use of Laboratory Animals” which was approved by the Institutional Animal Care and Use Committee of WIDMDC (Document: WHYXS/LL001-2014).

### Lipidomics

Samples were randomized before analysis. The preparation of serum samples and data acquisition followed the method published by Bird et al. (2011) with modifications. In general, serum (30 μL) lipids were extracted by adding 600 μL of 2:1 dichloromethane/methanol (DCM), vortexed at 4,000 rpm for 2 min, then adding 120 μL H_2_O and vortexed again for 1 min to complete the liquid-liquid extraction. Samples were incubated for 10 min at room temperature, and then centrifuged at 16,000 rcf for 10 min at 10°C. Aliquots of the lower lipid-rich DCM layer (370 μL) were transferred to new tubes and dried overnight on a Speed Vac. Dried extracts were reconstituted with 300 μL of acetonitrile/isopropanol/H2O (65:30:5, v/v/v) for instrumental analysis. For quality control (QC) purposes, 10 aliquots of commercial pooled mouse serum (Sigma Aldrich) were prepared identically and analyzed throughout the analysis sequences.

Lipidomics data was acquired in both positive and negative modes using a Waters ACQUITY UPLC system (Waters, Milford, MA) coupled with an LTQ Orbitrap Velos mass spectrometer (Thermo Fisher, San Jose, CA). Lipids were separated via a CSH C18 column (2.1 × 100 mm, 1.7 μm, Waters Technology) at 50°C with a flow rate at 0.25 mL min^−1^. In the binary reverse phase gradient for LC method, the mobile phase A was composed by H_2_O/acetonitrile (60:40) and the mobile phase B was composed by isopropanol/acetonitrile (90:10). Formic acid (0.1%) and ammonium formate (10 mM) were added to facilitate lipid ionization. A 10 μL injection volume was used and the data was collected from 120 to 2,000 m/z in positive and negative modes using the data dependent acquisition scan.

Peak alignment and normalization were performed using Progenesis QI (version 2.1, Waters, USA). The detected ions with retention time ≤1 min or ≥27 min, and peak width ≤0.1 min or ≥2.0 min, and mass range ≤250 m/z were excluded from further analysis. In addition, the highly variable ions with relative standard deviation (RSD) >30% in QC samples were also excluded. The “normalize to total intensity” mode, recommended by the software, was used to normalize the data.

Multivariate analysis, including principal component analysis (PCA), partial least squares discriminate analysis (PLS-DA) and orthogonal partial least squares discriminate analysis (OPLS-DA) were performed after Pareto-scaling and mean centering, with SIMCA 14.1 (Umetrics, Umeå, Sweden). The Variable Influence on Projection (VIP) value was used to evaluate the importance of the compounds for classification. Significance was determined using *t*-test in SAS 9.4 (SAS Institute Inc., Cary, NC). Peaks with VIP ≥ 1.0 were considered important to the differentiation of study groups, and *p* < 0.05 were considered significant. Lipids were identified through matching to databases including HMDB, Lipid Blast, and the in-house database based on exact mass and MS/MS fragmentation patterns.

### Targeted Metabolomics Analysis

Targeted analysis for serum metabolites was performed using the Absolute/IDQ™ p180 Kit (Biocrates Life Sciences AG, Innsbruck, Austria) with data acquired on an API 4000 (AB Sciex, Foster City, CA) triple quadrupole MS coupled with an Agilent 1200 HPLC (Santa Clara, CA). This kit enables quantitation to semi-quantification of 188 analytes, including 40 acylcarnitines, 20 amino acids, 19 biogenic amines, 76 phosphatidylcholines (PC), 14 lyso-phosphatidylcholines (LPC), 15 sphingomyelins (SM), and 1 hexose. Sample preparation and data acquisition were performed on randomized samples, according to the manufacturer's protocol and literature (Floegel et al., 2013), with LOD/LOQ indicated in supplementary material. Significant change in pairwise comparison was defined as *p* < 0.05 by *t*-test using SAS 9.4., and the importance was defined as VIP ≥ 1.0 via SIMCA.

### Cytokines

Serum cytokines were measured by using the Antibody G-5 Series array (RayBiotech, GA, USA) to profile 80 inflammatory cytokines simultaneously. Eight of ten samples per group were randomly selected by SAS 9.4, and then randomized before analysis. Experimental operations of blocking, sample loading, incubation, washing, and scan for detection of cytokine intensity were performed according to the manufacturer's instructions and literature (Stewart et al., 2016). The relative expression of each cytokine was normalized to the average value of positive controls in cytokine array slides. A heat map demonstrating the relative difference of each cytokine was generated using the manufacturer's threshold criteria for being up-regulated (≥1.5-fold) or down-regulated (≤-1.5-fold) for the vehicle treated WT mice, KB-, metformin-, and rosiglitazone- treated db/db mice vs. the vehicle treated db/db mice. Likewise, significance was calculated using Wilcoxon Rank Sum Test (*p* < 0.05).

### Pathway Analysis

GeneGo (MetaCore™) software (Encinitas, CA) was used for pathway analysis, and *p*-values and false discovery rates are provided for the top 12 pathways enriched from the “build your network” tool.

## Results

### KB Lowered Blood Glucose Without Body Weight Gain or Liver Mass Increase

**Figure 1:**
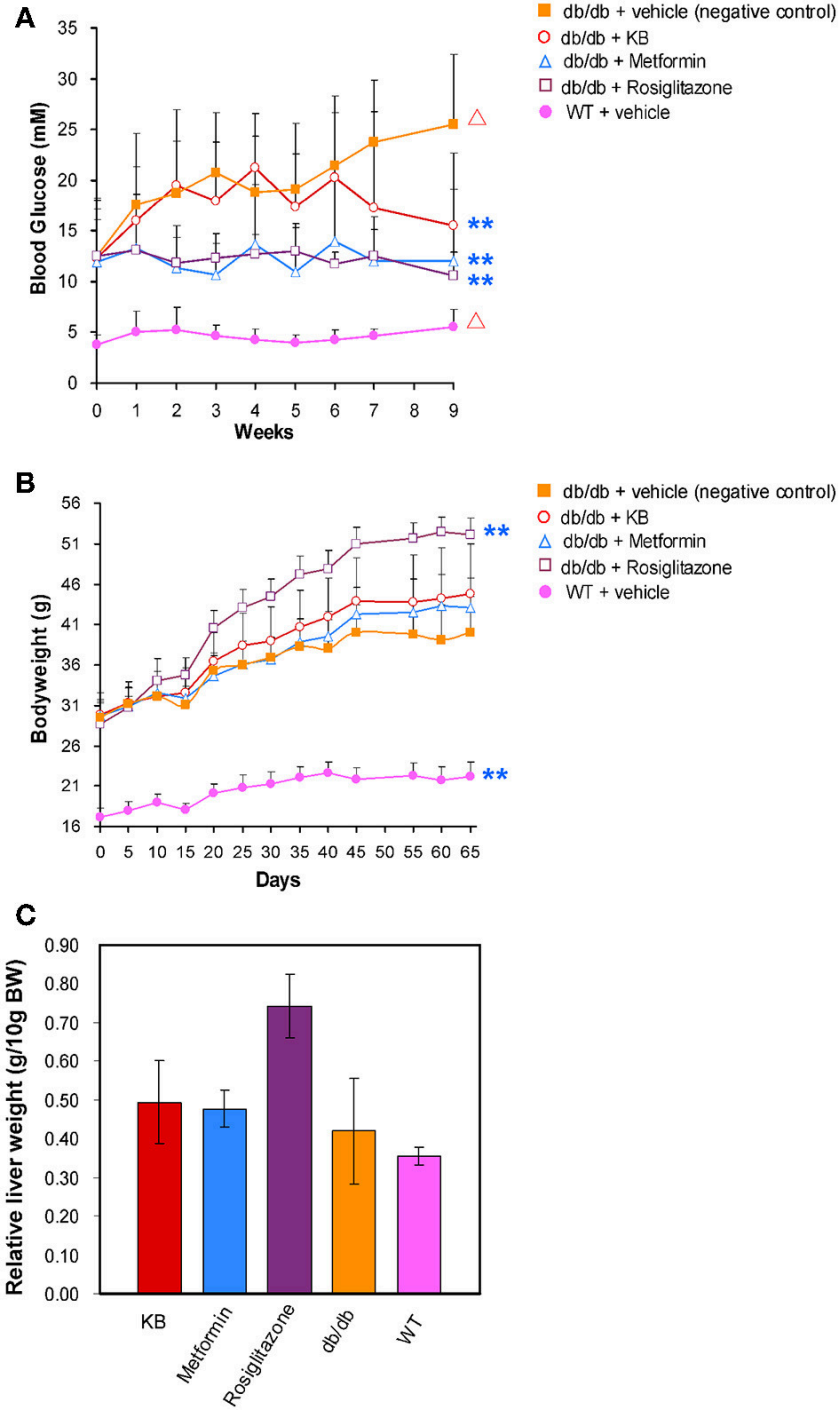
KB lowered blood glucose in db/db mice without bodyweight gain or hepatomegaly. **(A)** Effect of treatments on blood glucose. **(B)** Effect of treatments on body weight. **(C)** Effect of treatments on liver mass. Symbols: orange filled square, db/db + vehicle (negative control); pink filled circle, WT + vehicle; purple hollow square, db/db + rosiglitazone; blue triangle, db/db + metformin; red hollow circle, db/db + KB. ^**^p < 0.01 vs. vehicle + db/db; ^Δ^*p* < 0.05 vs. value at starting time point.

At 5 weeks old, fasting blood glucose in the vehicle-treated db/db mice (control) was approximately 3-fold higher than in the vehicle-treated WT mice (Figure 1A), demonstrating typical phenotypes of hyperglycemia. In addition, the 14-week-old mice showed a significant increase (*p* < 0.05) of blood glucose vs. the start-point at 5 weeks old, indicating an age associated blood glucose increase in db/db mouse model. After 9 weeks of treatment, the KB-treated db/db group (KB-db/db) showed lower blood glucose than control (*p* < 0.01) (Figure 1A). Also, treatment with KB successfully controlled the augment of blood glucose with age increase, as no significant difference in blood glucose was found between the start- and the end- points of the treatment. Both metformin and rosiglitazone presented outstanding hypoglycemic effects vs. control (*p* < 0.01). Furthermore, both metformin and rosiglitazone effectively inhibited the age-associated blood glucose increase in db/db mice (Figure 1A). After the treatment, no significant difference in blood glucose level was found among the KB-db/db, metformin-db/db, and rosiglitazone-db/db groups. It was noted that metformin and rosiglitazone had immediate hypoglycemic effects, which were effective in inhibiting the blood glucose increase from the first week of treatment. In contrast, the glucose-lowering effect of KB was slower and less potent, as the significant reduction in glucose was noticeable after 7 weeks of treatment.

At 5 weeks old, the bodyweight of db/db mice in control was 2-fold higher than that in the vehicle-treated WT mice (Figure 1B). Like blood glucose levels, the increase of bodyweight in db/db mice is also dependent on age (*p* < 0.05). Neither the KB- nor the metformin-db/db group showed significant weight gain or loss vs. control during or after the treatment. However, significant bodyweight gain was observed in the rosiglitazone-db/db group beginning at day 15, and the weight gain continued throughout the treatment. A mass increase in liver tissue was associated with rosiglitazone (Figure 1C), consistent with a previous study (Watkins, 2002). This side effect was not observed in the KB and metformin groups.

### Impact of KB Treatment on Lipid Profiles

**Figure 2:**
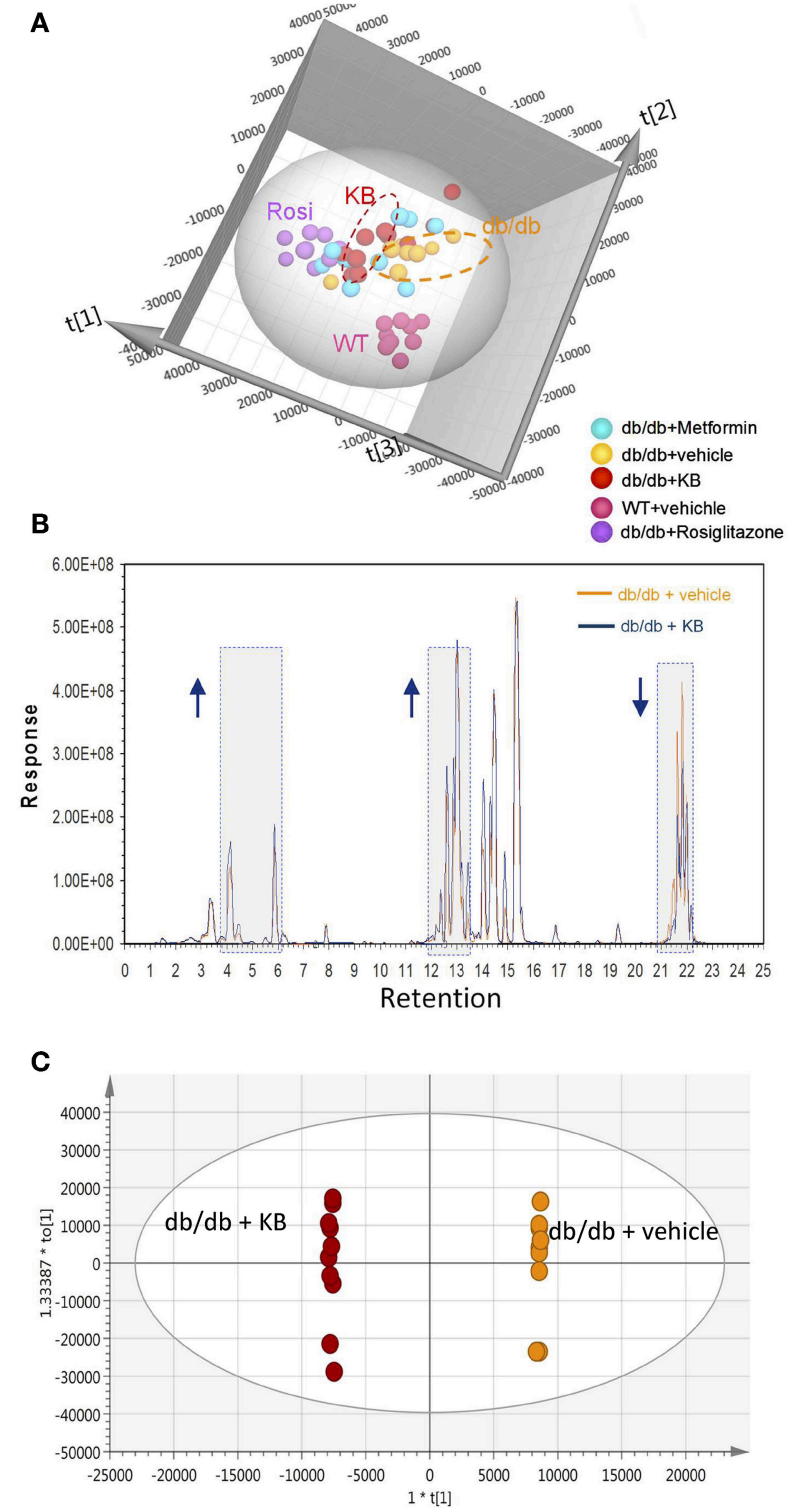
KB influenced circulating lipids in db/db mice. **(A)** Score scatter plot of the principal component analysis (PCA) for KB-, metformin-, rosiglitazone-, and vehicle-treated db/db mice, and vehicle-treated WT mice (data was acquired in positive mode). R2X (cum) = 0.939. **(B)** Overlap of typical base peak ion (BPI) chromatograms of KB- and the vehicle treated db/db (negative control) mice. Colors: dark blue line, KB +*db/db*; orange line, vehicle + *db/db*. Arrow up or down indicates the trend of signal change vs. negative control. **(C)** Score scatter plot of the supervised multivariate analysis (OPLS-DA) for KB +db/db vs. vehicle +db/db mice. R2X (cum) = 0.947, R2Y (cum) = 1, and Q2 (cum) = 0.764. Colors for **(A,C)**: Orange, vehicle+ db/db; pink, vehicle + WT; red, KB + db/db; blue, metformin + db/db; purple, rosiglitazone + db/db.

Circulating lipids were first investigated by broad-spectrum lipidomics. To obtain a comprehensive picture of the up- or down- regulation of lipids following treatments, LC-MS data were acquired by both positive and negative modes. Triglycerides (TG) and cholesterols (CHEL) were detected in positive mode, whereas most phosphatidylethanolamines (PE) and phosphatidylinositols (PI) were detected in negative mode (Table 1). Unsupervised multivariate analyses (PCA) for positive- (Figure 2A) and negative modes (Figure S1) are consistent in showing similar separation patterns for the investigated treatments, including vehicle-WT, vehicle-db/db, KB-db/db, metformin-db/db, and rosiglitazone-db/db. The vehicle-WT group was differentiated from the vehicle-db/db-group, indicating a shift of lipid metabolism in the db/db mice compared with WT mice. The drug response was evaluated by comparing with the vehicle-db/db group as control. KB treatment showed an obvious differentiation of the lipid profile from the control (Figure 2A). In the same direction as KB, the rosiglitazone treated group displayed a much greater differentiation from the control. However, the metformin-db/db group was clustered with the control, suggesting that metformin has limited influence on lipid metabolism.

**Table 1:** Serum lipids significantly responded to KB and the comparison with metformin and rosiglitazone.

**Accepted description**	**Detection methodology**	**Lipid class**	**KB-db/db vs. vehicle-db/db**			**Metformin-db/db vs. vehicle-db/db**			**Rosiglitazone-db/db vs. vehicle-db/db**		
			VIP^*^	*p*-value^**^	FC^***^	**VIP**	***p*-value**	**FC**	**VIP**	***p*-value**	**FC**
18:2 Cholesteryl ester	Pos	CHOL	7.0	0.02	−1.2	5.9	0.11	−1.1	3.2	0.24	−1.1
24-methylene-cholestan-3beta-ol	Pos	CHOL	1.2	0.01	−1.3	0.7	0.31	1.1	0.8	0.01	−1.3
lysoPC a C16:1	Bioc	LPC	1.4	<0.01	1.6	1.0	0.15	1.2	1.3	<0.01	3.0
lysoPC a C18:1	Bioc	LPC	1.2	0.03	1.5	0.9	0.16	1.3	1.3	<0.01	2.7
lysoPC a C20:3	Bioc	LPC	1.1	0.04	1.6	0.9	0.13	1.4	1.2	<0.01	2.8
lysoPC a C26:0	Bioc	LPC	1.4	<0.01	1.6	0.9	0.17	1.2	1.2	<0.01	2.2
lysoPC a C28:0	Bioc	LPC	1.2	0.02	1.3	1.2	0.05	1.2	1.1	<0.01	1.4
PC(15:1(9Z)/0:0)	Neg	LPC	1.4	<0.01	1.7	0.6	0.55	1.1	1.6	<0.0001	3.2
PC(16:1(9E)/0:0)	Neg	LPC	1.9	<0.01	1.7	0.7	0.60	1.1	2.2	<0.0001	3.2
PC(17:1(10Z)/0:0)	Neg	LPC	3.6	0.02	1.5	2.4	0.43	1.2	4.5	<0.0001	2.7
PC(18:1(6Z)/0:0)	Neg	LPC	5.9	0.02	1.5	3.6	0.49	1.1	7.5	<0.0001	2.7
PC(20:1(11Z)/0:0)	Neg	LPC	1.4	0.01	1.8	0.6	0.61	1.1	1.5	<0.0001	3.3
PC(16:1(9Z)/0:0)	Pos	LPC	1.9	0.02	1.5	1.0	0.29	1.2	2.4	<0.0001	2.8
PE(18:1(9Z)/0:0)	Neg	LPE	1.1	<0.01	1.5	0.4	0.21	−1.1	1.2	<0.0001	2.5
PE(16:0/0:0)	Neg	LPE	1.2	0.01	1.4	0.4	0.33	−1.1	0.6	0.07	1.3
MG(18:0/0:0/0:0)	Pos	MG	2.0	0.01	−1.2	1.8	0.05	−1.2	1.0	0.10	−1.1
PC aa C32:1	Bioc	PC aa	1.3	0.02	1.5	1.2	0.06	1.4	1.2	<0.01	4.2
PC aa C34:1	Bioc	PC aa	1.5	<0.01	1.5	1.3	0.03	1.4	1.3	<0.01	3.2
PC aa C34:3	Bioc	PC aa	1.4	<0.01	1.4	0.9	0.31	1.1	1.2	<0.01	2.1
PC aa C36:0	Bioc	PC aa	1.2	0.05	−1.2	0.9	0.28	1.1	1.2	<0.01	−1.5
PC aa C36:3	Bioc	PC aa	1.5	<0.01	1.6	1.1	0.09	1.2	1.3	<0.01	2.5
PC aa C38:0	Bioc	PC aa	1.2	0.03	−1.2	0.8	0.49	1.1	1.1	<0.01	−1.4
PC aa C38:3	Bioc	PC aa	1.4	<0.01	1.6	1.2	0.05	1.4	1.3	<0.01	2.3
PC aa C38:5	Bioc	PC aa	1.4	0.01	1.5	1.3	0.03	1.4	1.2	<0.01	2.1
PC aa C40:3	Bioc	PC aa	1.3	<0.01	1.3	0.9	0.37	1.1	1.3	<0.01	2.1
PC aa C40:4	Bioc	PC aa	1.4	<0.01	1.5	1.2	0.02	1.4	1.3	<0.01	2.3
PC(17:0/19:1)	Neg	PC aa	7.5	<0.01	1.4	5.4	0.17	1.2	10.0	<0.0001	2.6
PC(17:0/22:6)	Neg	PC aa	1.3	0.02	−1.3	0.6	0.80	1.0	1.5	<0.0001	−2.0
PC(18:0/20:3)	Neg	PC aa	11.0	<0.01	1.5	9.2	0.10	1.3	10.9	<0.0001	2.1
PC(18:0/20:4)	Neg	PC aa	8.5	<0.01	2.1	3.9	0.34	1.2	9.4	<0.0001	4.3
PC(18:0/22:5)	Neg	PC aa	2.1	0.03	1.2	2.7	0.10	1.2	1.5	0.02	1.2
PC(18:1/22:6)	Neg	PC aa	7.7	<0.01	1.6	5.3	0.19	1.2	5.6	<0.0001	1.8
PC(20:1/20:3)	Neg	PC aa	1.6	<0.01	2.5	0.8	0.23	1.3	2.0	<0.0001	6.1
PC(22:2/14:1)	Neg	PC aa	10.1	0.01	1.3	4.7	0.79	1.0	10.8	<0.0001	1.8
PC(24:1/14:1)	Neg	PC aa	2.2	0.01	1.4	1.1	0.35	1.1	3.1	<0.0001	2.6
PC(22:6/16:1)	Pos	PC aa	2.3	<0.01	1.5	1.3	0.38	1.1	1.7	<0.01	1.7
PC(18:2/17:0)	Pos	PC aa	1.1	0.02	1.4	0.9	0.12	1.3	1.0	<0.01	1.6
PC(22:6/17:0)	Pos	PC aa	1.8	0.03	−1.2	1.0	0.58	1.0	2.1	<0.0001	−1.8
PC(22:6/18:0)	Pos	PC aa	2.3	0.01	−1.3	1.6	0.11	−1.2	2.3	<0.0001	−2.3
PC(16:0/18:1)	Pos	PC aa	16.0	0.02	1.3	13.3	0.12	1.2	24.0	<0.0001	2.4
PC(17:0/18:0)	Pos	PC aa	1.3	0.01	1.2	0.9	0.31	1.1	0.9	0.0	1.2
PC(20:3/18:0)	Pos	PC aa	15.7	<0.01	1.5	11.3	0.11	1.3	16.1	<0.0001	2.2
PC(20:3/20:1)	Pos	PC aa	2.3	<0.01	2.5	0.7	0.42	1.2	3.0	<0.0001	7.0
PC(18:0/22:4)	Pos	PC aa	1.4	0.02	1.3	1.2	0.03	1.4	1.3	<0.0001	1.7
PC(18:1/18:0)	Pos	PC aa	8.0	0.04	1.3	5.8	0.17	1.2	13.3	<0.0001	2.7
PC(18:2/20:0)	Pos	PC aa	2.4	0.02	1.3	1.1	0.62	1.1	4.1	<0.0001	2.9
PC ae C36:1	Bioc	PC ae	1.2	0.02	1.3	1.0	0.10	1.3	1.3	<0.01	2.4
PC ae C34:2	Bioc	PC ae	1.4	<0.01	−1.3	0.9	0.49	−1.1	1.0	0.01	−1.3
PC ae C34:3	Bioc	PC ae	1.3	<0.01	−1.3	0.7	0.66	1.0	1.1	<0.01	−1.4
PC ae C36:2	Bioc	PC ae	1.4	<0.01	−1.3	1.0	0.23	−1.1	1.2	<0.01	−1.6
PC ae C38:2	Bioc	PC ae	1.1	0.03	−1.2	1.1	0.07	−1.2	1.1	<0.01	−1.4
PC ae C38:3	Bioc	PC ae	1.3	0.01	1.2	0.9	0.18	1.1	1.0	0.01	1.3
PC ae C38:6	Bioc	PC ae	1.2	0.01	−1.2	0.8	0.80	1.0	1.1	<0.01	−1.4
PC ae C40:6	Bioc	PC ae	1.5	<0.01	−1.2	1.0	0.18	1.1	1.3	<0.01	−1.8
PC ae C42:1	Bioc	PC ae	1.3	0.01	1.3	1.2	0.00	1.4	1.0	0.02	1.3
PC ae C42:4	Bioc	PC ae	1.1	0.04	−1.1	0.5	0.25	1.1	1.1	<0.01	−1.4
PC ae C44:5	Bioc	PC ae	1.2	0.03	−1.2	0.7	0.77	1.0	1.3	<0.01	−1.6
PC(O-16:0/22:6)	Neg	PC ae	1.5	0.02	−1.3	1.3	0.24	−1.1	1.1	0.01	−1.3
PC(O-16:0/22:6)	Pos	PC ae	2.1	0.02	−1.3	1.2	0.59	−1.1	1.3	0.03	−1.3
PC(O-16:0/18:2)	Pos	PC ae	1.6	<0.01	−1.4	0.7	0.82	−1.0	0.7	0.09	−1.2
PC(O-20:0/20:4)	Pos	PC ae	1.0	0.01	−1.3	0.4	1.00	1.0	0.8	<0.01	−1.3
PC(O-20:0/18:2)	Pos	PC ae	1.1	0.02	−1.3	0.2	0.28	1.1	0.8	<0.01	−1.3
PE(O-18:0/18:2)	Neg	PE	1.5	0.01	−1.6	0.8	0.56	−1.1	1.4	<0.01	−2.4
PE(O-18:0/22:4)	Neg	PE	1.1	0.03	−1.4	0.5	0.88	1.0	1.0	<0.01	−2.1
PE(O-18:0/22:6)	Neg	PE	4.9	<0.01	−1.6	4.5	0.04	−1.34	4.3	<0.0001	−2.6
PE(O-20:0/18:2)	Neg	PE	1.1	0.03	−1.4	0.5	0.77	−1.04	1.1	<0.01	−2.3
PE(O-20:0/20:4)	Neg	PE	1.7	0.04	−1.3	0.9	0.73	−1.04	1.6	<0.01	−1.9
PE(O-20:0/22:6)	Neg	PE	2.0	0.01	−1.4	1.9	0.04	−1.29	2.0	<0.0001	−2.6
PE(P-18:0/18:2)	Neg	PE	1.2	0.03	−1.4	0.8	0.32	−1.2	1.2	<0.01	−2.3
PE(P-18:0/20:4)	Neg	PE	2.7	0.05	−1.3	1.6	0.61	−1.1	2.8	<0.01	−1.8
PE(P-18:0/22:4)	Neg	PE	1.7	<0.01	−1.6	1.1	0.23	−1.2	1.5	<0.0001	−2.9
PE(P-18:0/22:6)	Neg	PE	2.4	0.03	−1.3	2.5	0.08	−1.2	2.8	<0.0001	−2.4
PE(P-18:0/22:6)	Pos	PE	1.1	<0.01	−1.4	0.8	0.05	−1.2	1.0	<0.0001	−2.1
PE(O-20:0/22:6)	Pos	PE	1.1	0.01	−1.4	0.8	0.05	−1.2	0.9	<0.01	−1.8
PE(22:0/22:1)	Pos	PE	1.1	<0.01	−1.5	0.7	0.07	1.3	0.3	0.13	−1.2
PE-NMe2(16:0/18:1)	Neg		2.0	0.01	1.4	1.3	0.20	1.2	3.2	<0.0001	3.0
PI(16:0/20:4)	Neg	PI	4.1	<0.01	1.4	3.4	0.15	1.2	2.2	<0.01	1.3
PI(18:0/18:2)	Neg	PI	2.4	0.01	−1.4	3.1	0.02	−1.4	1.4	0.02	−1.3
PI(18:0/20:3)	Neg	PI	2.8	0.01	1.9	1.8	0.89	1.0	4.6	<0.0001	2.7
PI(18:0/20:3)	Neg	PI	3.3	0.02	1.4	1.5	0.45	1.2	3.8	<0.0001	4.4
PI(18:1/18:2)	Neg	PI	2.0	<0.01	1.3	0.9	0.89	1.0	1.6	<0.01	1.5
PI(18:1/20:4)	Neg	PI	5.0	<0.0001	1.9	2.7	0.2	1.3	3.9	<0.0001	2.4
PI(18:4/20:1)	Pos	PI	1.5	0.00	1.7	0.6	0.11	1.3	1.1	<0.0001	2.1
PS(19:1/22:2)	Neg	PS	1.6	0.01	1.3	1.1	0.23	1.1	2.1	<0.0001	2.0
PS(22:4/19:1)	Neg	PS	2.3	<0.01	1.2	1.0	0.85	1.0	2.5	<0.0001	1.5
PS(P-20:0/15:0)	Pos	PS	2.7	0.04	−1.5	1.6	0.30	−1.2	1.6	0.10	−1.4
PS(O-20:0/17:1)	Pos	PS	3.8	0.03	−1.3	3.0	0.10	−1.2	2.8	0.01	−1.4
SM (OH) C16:1	Bioc	SM	1.5	<0.01	−1.3	1.1	0.07	1.2	1.2	<0.01	−1.8
SM C20:2	Bioc	SM	1.4	<0.01	1.7	1.3	0.01	1.5	0.9	0.01	1.5
SM(d18:1/24:1)	Neg	SM	4.5	<0.01	1.2	5.5	0.05	1.2	6.5	<0.0001	1.9
SM(d16:1/18:1)	Pos	SM	1.0	0.05	−1.1	0.6	0.67	−1.0	0.9	<0.01	−1.3
SM(d18:2/22:0)	Pos	SM	1.5	0.03	−1.3	1.3	0.05	−1.2	1.0	0.01	−1.3
SM(d17:1/24:0)	Pos	SM	1.5	<0.01	−1.2	1.1	0.05	−1.1	1.6	<0.0001	−2.0
SM(d18:1/24:0)	Pos	SM	2.1	<0.01	−1.3	1.4	0.09	−1.1	1.8	<0.0001	−1.7
TG(18:2/20:5/22:6)	Pos	TG	1.0	<0.01	−2.4	0.6	0.11	−1.3	0.8	<0.0001	−7.8
TG(20:4/22:6/22:6)	Pos	TG	1.1	<0.01	−2.0	0.4	0.78	−1.0	1.0	<0.0001	−6.0
TG(20:2/22:6/22:6)	Pos	TG	3.4	<0.01	−2.2	1.9	0.12	−1.3	2.6	<0.0001	−4.4
TG(20:1/20:5/22:6)	Pos	TG	4.8	<0.01	−1.8	2.2	0.41	−1.1	3.9	<0.0001	−3.2
TG(18:0/18:3/22:6)	Pos	TG	10.6	0.04	−1.7	9.1	0.12	−1.4	9.9	<0.01	−3.8
TG(18:4/20:1/22:4)	Pos	TG	5.9	0.01	−1.7	4.5	0.08	−1.4	4.7	<0.01	−2.6
TG(18:1/18:0/20:3)	Pos	TG	1.1	0.03	−1.6	1.1	0.02	−1.6	1.0	<0.01	−2.8
TG(20:1/18:0/20:5)	Pos	TG	1.6	0.02	−1.6	1.4	0.03	−1.5	1.5	<0.01	−2.8
TG(20:5/20:5/18:0)	Pos	TG	1.1	0.03	−1.7	1.2	0.01	−1.9	1.0	<0.01	−3.2
Coenzyme Q9	Pos	ubiquinone-9	1.0	<0.01	−1.5	0.7	0.05	1.3	3.2	0.3	−1.1

**Figure 3:**
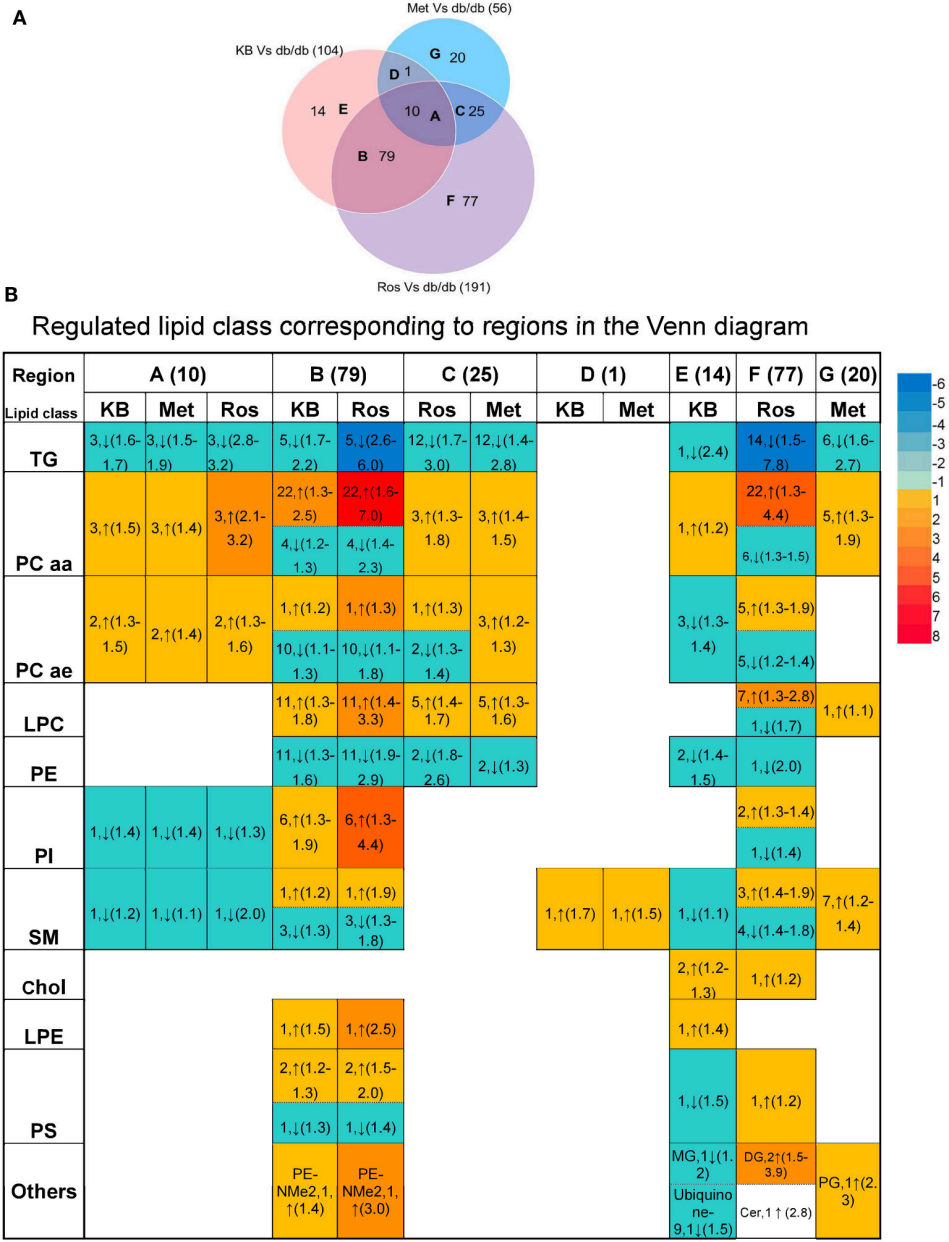
Comparison of lipids responding to KB, rosiglitazone, and metformin treatment. **(A)** Venn diagram of altered lipids following treatment with KB, rosiglitazone and metformin in db/db mice. Lipids which differentiated treatment groups (i.e., KB-db/db, rosiglitazone-db/db, and metformin-db/db) from control group (i.e., vehicle-db/db) satisfying VIP ≥ 1.0 and *p* < 0.05 were defined as altered. The capital letters inside each circle of the diagram represent the overlap of altered lipids following each respective treatment; and the digit next to the capital letters indicate the number of lipid species that overlap. Digits in parentheses outside the Venn diagram represent the total number of altered lipids from each treatment. **(B)** Classes of the lipids corresponding to different regions of the Venn diagram. Arrow direction indicates up- or down-regulation of the lipid species. The digit before the arrow indicates the number of lipid species identified in each class, and the numbers inside the parentheses indicate fold-change from low to high. Colors from blue to red indicate the fold-change magnitudes.

Detection methodology: Pos and Neg, identification of lipids from broad spectrum lipidomics in either positive or negative mode; Bioc, quantitation of lipids through targeted lipidomics using Biocrates p180 kit. Lipid class: CHOL, cholesterol; LPC, lysophosphatidylcholine; LPE, lysophosphatidylethanolamine; MG, monoglyceride; PC aa, diacyl-phosphatidylcholine; PC ae, alkylacyl-phosphatidylcholine; PE, phosphatidylethanolamine; PI, phosphatidylinostitol; PS, phosphatidylserine; SM, sphingomyelin; TG, triglyceride. VIP^*^, variable influence on projection; FC^***^, fold change (compared to “vehicle-db/db” based on mean); p-value^**^, significance examination via t-test. Cut-off criteria for significance of lipids responding to treatment: VIP ≥ 1.0, and p < 0.05. Gray space: No change; Red: increase; Blue: decrease. Table 1 lists the lipids that responded to KB. The specific lipid profiles responding to metformin and rosiglitazone are listed in Tables S1, S2 and Figure 3.

The overlap of the base peak chromatogram (BPC) from the KB-db/db and the vehicle-db/db samples in positive mode (Figure 2B) exhibits most signal differences in three retention-time regions. For the C_18_ column used, we expect the down-regulated signals at the region of 21.0–22.5 min to be associated with non-polar lipids, such as triglycerides, cholesterols, and cholesterol esters (Bird et al., 2011; Fauland et al., 2011). The up-regulated signals in the region of 13.0–15.0 min are attributed to phospholipids, while the signals in the region of 3.5–6.5 min are attributed to lysophospholipids and other hydrosoluble small lipid-related metabolites.

An additional aliquot of serum from the same animals was measured by a targeted approach using the Biocrates p180 kit, in which 106 lipid species were visualized by PCA. The pattern of PCA score scatter plot for this targeted approach (Figure S3) is very similar to that of broad spectrum lipidomics (Figure 2A and Figure S1), demonstrating consistent results between targeted and untargeted approaches.

Supervised pairwise comparison between drug-treated group (KB-db/db, metformin-db/db, and rosiglitazone-db/db) and control (vehicle-db/db) was performed by OPLS-DA for both the untargeted and targeted lipidomics data (Figure 2C and Figures S2, S4). Compounds satisfying VIP ≥ 1.0 and *p* < 0.05 were considered as treatment-responsive lipids and summarized in Table 1 and Figure 3. As shown in the Venn diagram (Figure 3A), 104 lipids were identified as the KB-perturbed lipids to differentiate the KB-db/db group from the vehicle-db/db control. Likewise, 191 lipids were perturbed by rosiglitazone; while only 56 lipids were perturbed by metformin. Overall, 75% of altered lipids were in common between KB and rosiglitazone. However, very few of these lipids were influenced by metformin.

The drug-responsive lipid class corresponding to different regions of the Venn diagram is shown in Figure 3B. KB treatment significantly decreased triglycerides (TG), phosphatidylethanolamines (PE), monoglycerides (MG), cholesterol, cholesteryl ester, coenzyme Q9, as well as most of sphingomyelin (SM), and alkylacyl-phosphatidylcholines (PCae) species, accompanied by an increase of diacyl-phosphatidylcholine (PCaa) and lysophosphatidylcholine (LPC) and phosphatidylinositol (PI) (Table 1 and Figure 3B). It is interesting to find that phosphatidyl-dimethylethanolamine (PE-NMe2), the intermediate of PC biosynthesis from PE via the phosphatidylethanolamine N-methyltransferase (PEMT) pathway (Gibellini and Smith, 2010; Cole et al., 2012), was elevated 1.4-fold, indicating the activation of this pathway.

Rosiglitazone-db/db vs. control and KB-db/db vs. control showed consistent regulated directions for all lipid classes (Table 1 and Figure 3B), but rosiglitazone induced a dramatically greater magnitude of fold-change and impacted more lipid species in each class.

Metformin caused less perturbation in lipid species and alteration of magnitude than KB and rosiglitazone (Figure 3B, Table 1, and Table S1). Compared with phospholipids, TG seems more responsive to metformin treatment. Among the few metformin-influenced lipids, TG, LPC, PCaa, and PE were up- or down- regulated in the same direction as KB and rosiglitazone. Since metformin, rosiglitazone, and KB were effective in regulating blood glucose of db/db mice in different levels, changing of these lipid classes might be related to glucose homeostasis.

### Influence of KB on Acylcarnitine Profiles Compared to Rosiglitazone and Metformin

The influences of KB, metformin, and rosiglitazone on acylcarnitine profile were studied using the Biocrates p180 kit. A total of 21 acylcarnitines were measured in a semi-quantitative manner (Table 2), while 19 species were lower than the limit of detection. Carnitine (C0) and acetylcarnitine (C2) were found to be the most abundant species in the acylcarnitine profile, with 10- to 100-fold higher abundance than those with longer carbon chains. The vehicle-db/db mice generally had lower serum acylcarnitine levels than the vehicle-WT mice (Table 2). Compared to the control (vehicle-db/db), the KB-db/db group showed moderate increases of the C18, C16, C14, C3, and C2 acylcarnitines with/without hydroxyl- or unsaturated- fatty acid conjugates. Rosiglitazone-db/db vs. control displayed significantly increased acylcarnitines, as well as more perturbed acylcarnitine species with greater fold-change magnitudes than KB-db/db vs. control. Metformin appeared to work in a different way, as the short-chain acylcarnitines, such as C0, C2, C4, C5, and C6 were decreased compared to control.

**Table 2:** Up- and down- changed acylcarnitines in response to different treatments.

**Acylcarnitines**	**Range of value (nM or μM^**Δ**^)**	**KB-db/db vs. vehicle-db/db**		**Met-db/db vs. vehicle-db/db**		**Ros-db/db vs. vehicle-db/db**		**Vehicle-WT vs. vehicle-db/db**	
		FC^*^	*p*-value^**^	**FC**	***p*-value**	**FC**	***p*-value**	**FC**	***p*-value**
C0	^Δ^9.95–31	−1.25	0.11	−1.42	0.03	−1.24	0.11	−1.16	0.38
C14	81–427	1.06	0.49	−1.12	0.21	1.39	0.02	2.18	0.00
C14:1	80–294	1.19	0.03	−1.09	0.29	1.82	<0.001	1.27	0.04
C14:1-OH	12–37	1.20	0.04	−1.01	0.87	1.32	0.05	1.51	0.01
C14:2	15–44	−1.06	0.57	−1.24	0.06	1.09	0.48	1.27	0.04
C16	295–1010	1.07	0.31	1.09	0.22	1.08	0.40	1.85	0.00
C16-OH	10–45	1.34	0.02	1.27	0.01	1.45	<0.001	2.14	0.00
C16:1	67–298	1.35	0.01	−1.02	0.84	2.07	<0.001	1.23	0.11
C16:1-OH	11–39	1.16	0.16	1.01	0.95	1.37	0.02	1.52	0.01
C16:2	12–65	−1.03	0.79	−1.26	0.13	−1.20	0.24	1.69	0.00
C18	40–235	−1.17	0.02	−1.01	0.89	−1.31	0.00	2.16	0.00
C18:1	210–588	1.21	0.02	1.00	0.97	1.29	0.03	1.27	0.05
C18:2	37–286	−1.06	0.65	−1.25	0.12	−1.57	0.01	1.56	0.01
C2	^Δ^10.6–55.5	1.33	0.02	−1.35	0.03	1.07	0.50	1.42	0.01
C3	119–1010	1.12	0.44	−1.18	0.28	1.00	0.99	1.76	0.04
C4	275–1330	1.25	0.06	−1.29	0.03	1.53	0.00	−1.14	0.25
C3-DC (C4-OH)	100–441	1.44	0.02	1.11	0.50	1.26	0.12	1.34	0.03
C5	82–231	−1.07	0.44	−1.30	0.01	−1.05	0.53	1.21	0.16
C6 (C4:1-DC)	33–206	1.00	0.99	−1.41	0.02	1.21	0.18	2.26	0.00

*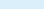 Decreased significantly 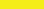 Increased significantly 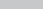 Not changed Acylcarnitine C10, C10:1, C10:2, C12, C12-DC, C12:1, C14:2-OH, C16:2-OH, C3-OH, C3:1, C4:1, C5-M-DC, C5-OH (C3-DC-M), C5:1, C5:1-DC, C5-DC(C6-OH) C6:1, C7-DC, C8, C18:1-OH, and C9 were not listed as they were out of detection limit. “Met” means “metformin,” “Ros” means “rosiglitazone,” and WT means “wild type mice.” Fold change (FC) (drug-treated groups, or wild type) was compared to “vehicle-db/db” based on mean. p-value was calculated by t-test. Cut-off criterion: ^*^FC > 1.5, or ^**^p < 0.05. Range of value: concentration range of analyte across investigated samples. ^Δ^the unit for acyl carnitine C0 (carnitine) and C2 (acetyl carnitine) are μM, others are nM*.

Unlike for lipids and acylcarnitines, perturbation of amino acids and biogenic amines by KB was minor in db/db mice (Table S3). Results for hexose were not reported in the Biocrates section, because the data did not pass the QC in pool samples.

### KB Reduced Chronic Inflammation in db/db Mice

**Figure 4:**
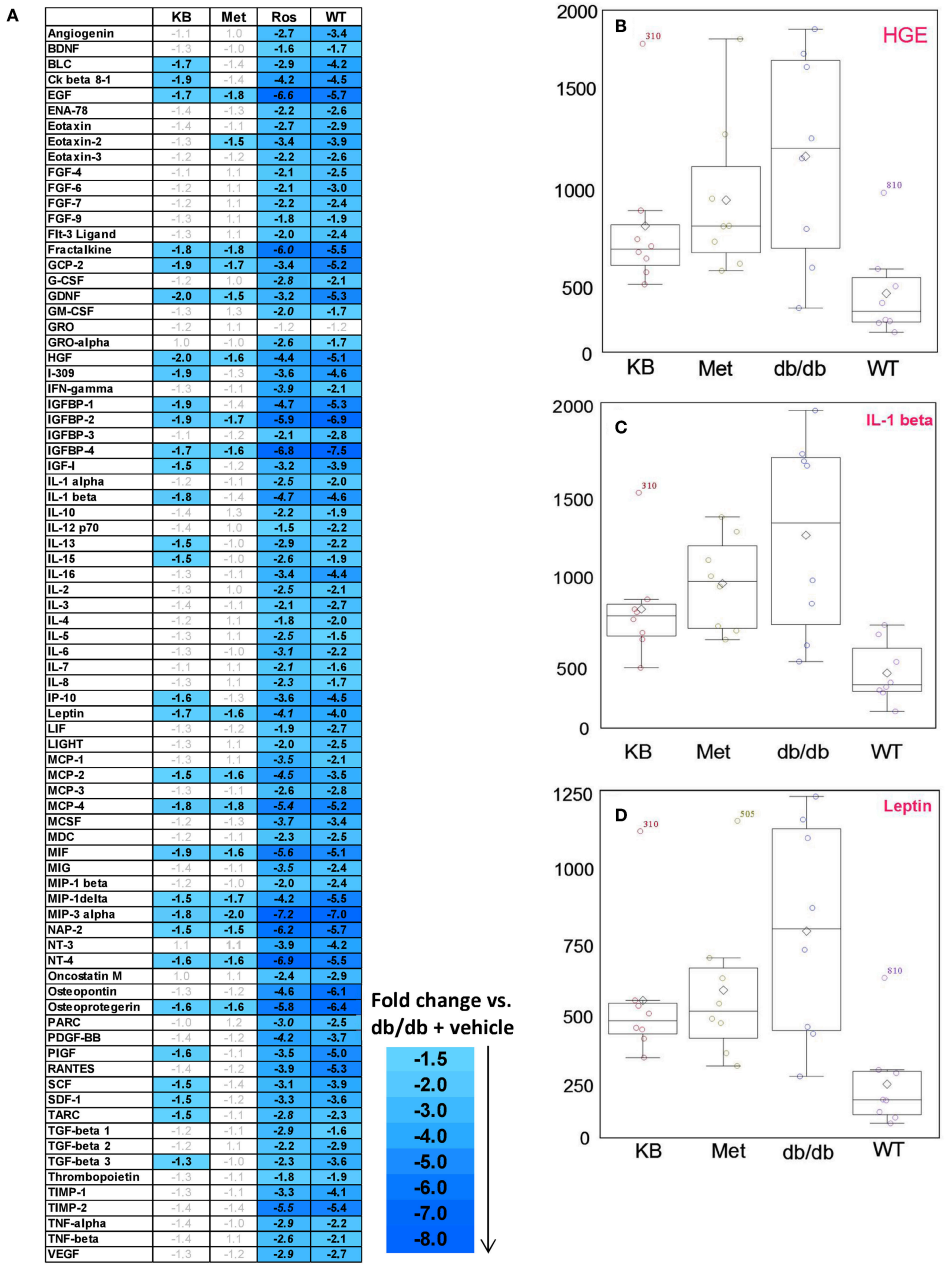
Influence of KB, metformin, and rosiglitazone on chronic inflammation in db/db mice. **(A)** The color-graded table demonstrates relative fold-change of cytokines for KB-, metformin-, and rosligtazone-treated db/db mice, as well as the vehicle-WT group, all normalized to the vehicle-db/db group (control). The fold changes are based on the median of biological replicates in each group (*n* = 8). Threshold criteria for being up-regulated (≥1.5-fold) or down-regulated (≤-1.5-fold) are according to manufacturer's guidelines (RayBiotech). A negative fold change means the cytokine level is lower than in the control (vehicle-db/db group). **(B–D)** Box plots for cytokines of interest in KB-db/db and metformin-db/db mice: **(B)** HGE; **(C)** IL-1β; **(D)** Leptin. KB, Met, and Rosi indicate KB-, metformin-, and rosiglitazone treated db/db mice, respectively; WT indicates vehicle-treated wild type mice.

Eighty circulating inflammatory markers were profiled in a separate aliquot of the same serum samples. As shown in Figure 4, the vehicle treated db/db mice (control) had overall higher cytokine levels than the vehicle-treated WT group, consistent with previous studies (Esser et al., 2014). After treatment, the KB-db/db mice showed reduced levels of 29 inflammatory markers vs. control (Figure 4). The rosiglitazone-db/db group showed sharp decreases of all cytokines vs. control. For several cytokines, this reduction was even greater than that of vehicle-WT vs. vehicle-db/db. The metformin-db/db group showed moderate to no down-regulation in measured cytokines vs. control, reducing only 15 cytokines. Both metformin and KB moderately regulated the chemokines, IGF-1, leptin, and growth factor hormones; however, metformin did not decrease any of the interleukins (Table 3 and Table S3).

**Table 3:** Down-regulated cytokines in response to KB treatment in db/db mice.

**Cytokine**	**Alias**	FC^**^	*p*-value^*^
^▴^GDNF	Glial cell line-derived neurotrophic factor	−2	0.21
^▴^HGF	Hepatocyte growth factor	−2	0.25
^▴^IGFBP-2	Insulin like growth factor binding protein 2	−1.9	0.25
^▴^GCP-2	Granulocyte chemotactic protein 2, or CXCL6	−1.9	0.29
I-309	CCL 1	−1.9	0.33
^▴^IGFBP-1	Insulin like growth factor binding protein 1	−1.9	0.39
^▴^MIF	Macrophage migration inhibitory factor	−1.9	0.44
Ck beta 8-1	CCL23	−1.9	0.51
IL-1 beta	Interleukin 1 beta	−1.8	0.15
^▴^Fractalkine	CX3CL1	−1.8	0.39
^▴^MCP-4	CCL13, monocyte chemoattractant protein 4	−1.8	0.39
MIP-3 alpha	CCL19	−1.8	0.39
EGF	Epidermal growth factor	−1.7	0.21
^▴^IGFBP-4	Insulin like growth factor binding protein 4	−1.7	0.21
^▴^Leptin		−1.7	0.29
BLC	CXCL13	−1.7	0.33
^▴^IP-10	CXCL10	−1.6	0.33
^▴^Osteoprotegerin	Tumor necrosis factor receptor superfamily member 11B	−1.6	0.33
NT-4	neorotrophin-4	−1.6	0.44
PIGF	Placental growth factor	−1.6	0.58
^▴^TARC	Thymus and activation regulated chemokine, CCL17	−1.5	0.01
^▴^IL-13	Interluekin-13	−1.5	0.18
^▴^IGF-I	Insulin-like growth factor 1	−1.5	0.33
MIP-1delta	Macrophage inflammatory protein, CCL3 AND CLL4	−1.5	0.44
NAP-2	CXCL7	−1.5	0.64
^▴^IL-12 p70	Interleukin 12	−1.4	0.03
Flt-3 Ligand	Fms-like tyrosine kinase 3, fetal liver kinase-2	−1.3	0.05
^▴^IL-4	Interluekin-4	−1.2	0.03

“*In response to KB treatment” indicates cytokines significantly change in KB-treated db/db mice vs. vehicle-treated db/db mice (negative control). Fold change (FC) is compared to “vehicle-db/db” based on median. Wilcoxon rank-sum test was used for calculating the p-value. Threshold for significance:*

* FC > 1.5, or

** *p < 0.05*.

**Figure 5:**
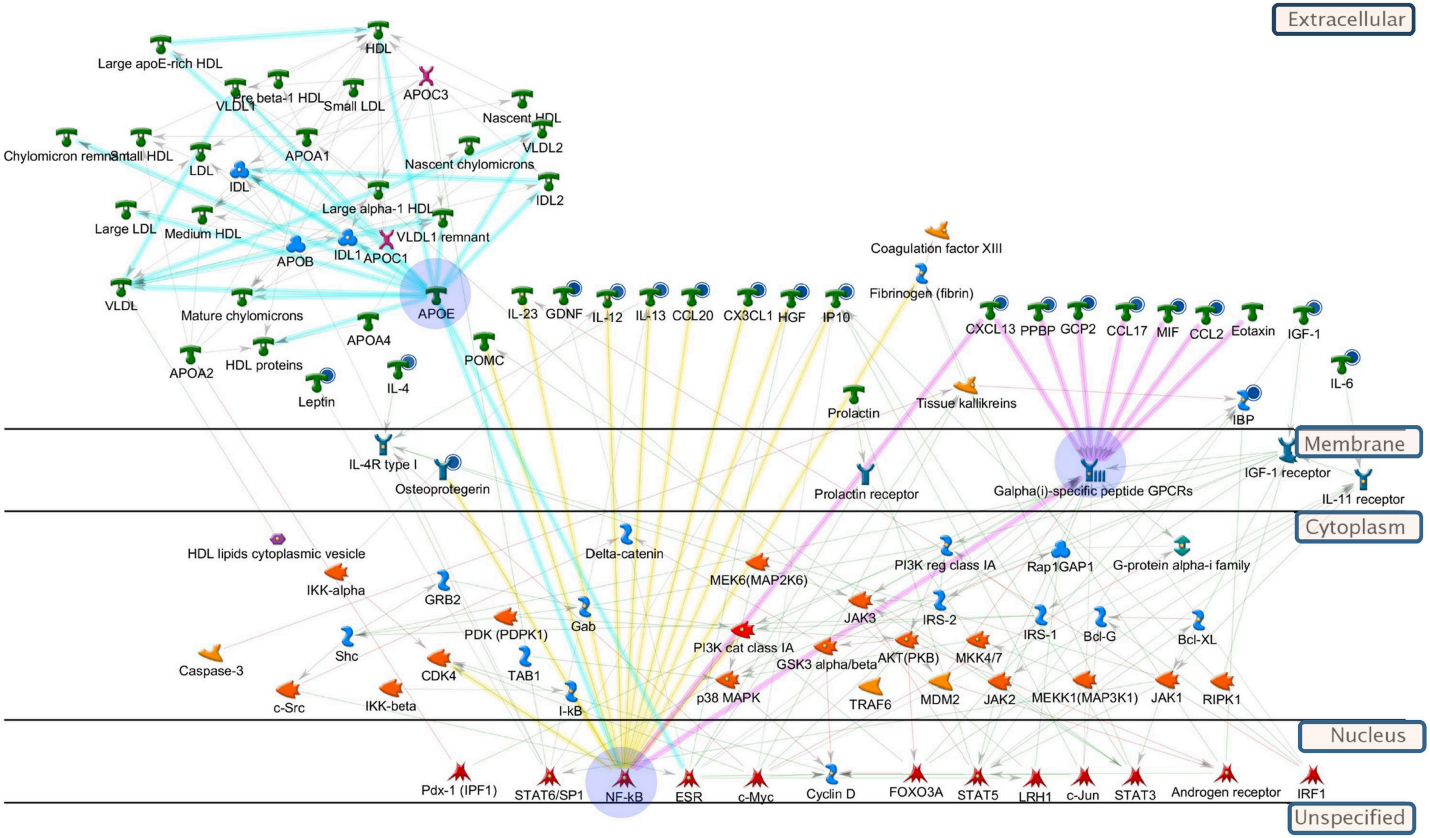
Pathway analysis of biomolecules differentiating vehicle-treated vs. KB-treated db/db mice. All-important metabolites, lipids, and cytokines were included in the enrichment analysis (GeneGo, MetaCore). The highlighted blue circles indicate the cytokines (or metabolites) being reduced by KB. Yellow highlighted linkages: cytokines regulated by up-stream transcription factors, e.g., NF-κB. Pink highlighted linkages: reduction of cytokines regulate down-stream receptors, Galpha(i)-specific peptide GPCRs, to influence metabolism. Light blue-highlighted linkages: apolipoprotein (e.g., APOE) regulated by transcription factors. Explanation of pathway map symbols can be found at https://portal.genego.com/.

▴ *represents that the cytokines were included in the generated pathway by GeneGo, shown as Figure 5*.

## Discussion

We used a multi-*omics* approach to investigate the efficacy of KB, a natural product purified from the TCM herb Lycii Cortex, on T2DM by means of the db/db diabetic mouse model. Compared to the vehicle-treated db/db mice (control), KB (1) attenuated blood glucose without bodyweight gain or hepatomegaly; (2) regulated lipid and energy metabolic pathways toward homeostasis; and (3) reduced chronic inflammation. This *in vivo* study has provided direct evidence that KB is the major bioactive molecule from LyC that intervenes early diabetes. This research also provides new insight about the influence of low-dose metformin and rosiglitazone on lipid metabolism and circulating inflammatory markers in the diabetic mouse model.

Measurement of fasting blood glucose reduction is the gold standard to evaluate efficacy of anti-T2DM drugs. Even though the dosages used in this study are lower than the levels commonly used for therapeutic purposes (Fujita et al., 2005; Chodavarapu et al., 2013; Adam et al., 2016), metformin and rosiglitazone still presented significant effects in attenuating blood glucose increase (Figure 1). Rosiglitazone is reported to associate with adverse effects such as bodyweight gain and hepatomegaly (Watkins, 2002; Ahmadian et al., 2013). In our animal study, we also observed weight-gain and the increased liver tissue mass in the rosiglitazone-treated db/db mice compared to controls; whereas these adverse effects were not observed in the KB-db/db mice, despite the hypoglycemic effect of KB being much slower and less acute than rosiglitazone and metformin (Figure 1). We postulated that KB might indirectly regulate cellular glucose update or consumption, so that the immediate hypoglycemic effect was not as outstanding as those first-line drugs. However, since T2DM is a complicated disease with systemic dysfunctions underlying the symptom of hyperglycemia, KB may work via correction of one or more of the metabolic disorders, thus gradually helping the body to recover homeostasis.

Lipidomics revealed that KB reduced serum TG levels (Table 1 and Figure 3), consistent with results found in the metformin- and rosiglitazone-db/db mice; although to different magnitudes. A decrease in TG is expected to be associated with improvement of lipid homeostasis by several means, including (1) facilitating lipogenesis rather than adipocyte lipolysis (Ahmadian et al., 2013; Frühbeck et al., 2014; Kwon et al., 2015), (2) improving lipoprotein composition by reducing low-density lipoprotein (LDL) and increasing high-density lipoproteins (HDL) (Ahmadian et al., 2013; Geerling et al., 2014), and (3) accelerating lipid clearance and fatty acid oxidation (Geerling et al., 2014; Kwon et al., 2015). In addition to alterations in TG, we found that KB substantially increased circulating PC and LPC but decreased PE (Table 1 and Figure 3). There are mainly two *in vivo* pathways for synthesizing PC (Gibellini and Smith, 2010; Cole et al., 2012). One is the “Kennedy pathway,” which starts from choline occurring in all nucleated cells, and the other is the “PEMT pathway” which mainly occurs in the liver *via three* sequential methylations of PE by the enzyme phosphatidylethanolamine N-methyltransferase (PEMT) (Gibellini and Smith, 2010; Cole et al., 2012). KB was found to increase PE-NMe2 (16:0/18:1) (Table 1), suggesting it may influence the PEMT pathway and facilitate PC biosynthesis from PE. Because PC is required for lipoprotein assembly and secretion, especially for VLDL and HDL, this process is considered helpful for lipid secretion, transportation and clearance (Cole et al., 2012). Furthermore, the abundant PCs [such as PC (18:0/18:1) and PC (16:0/18:1)] which were found to respond to KB treatment in this study (Table 1) are endogenous PPAR δ or -á ligands, and have been proved to regulate glucose and lipid homeostasis (Chakravarthy et al., 2009; Lamaziere and Wolf, 2010; Liu et al., 2013). The increase of LPC (Table 1 and Figure 3B) by KB may result from either PC hydrolysis via enzymes from the phospholipase A2 superfamily, or cholesterol esterification which was catalyzed by the lecithin-cholesterol acyltransferase (LCAT) to transfer fatty acid moieties from position sn-2 of PC to circulating cholesterol to form cholesterol esters (Schmitz and Ruebsaamen, 2010). However, further work is needed to define which pathway was activated by KB to increase the LPC. The role of LPC in T2DM is still controversial; some researchers consider LPCs as a pro-inflammatory factor associated with atherosclerosis and cardiovascular disease comorbidities (Huang et al., 1999), while others believe LPC may be beneficial for glucose and metabolic homeostasis, and not related to insulin resistance (Yea et al., 2009; Klingler et al., 2016). Our results support the second argument that LPC facilitates metabolic homeostasis and anti-inflammation (Figure 4), although the mode of action has not yet been clearly defined.

Parallel comparisons with the first-line anti-T2DM drugs indicated that KB works in a similar way to rosiglitazone in regulating lipid profiles (Figure 3), although rosiglitazone was far more potent. Both KB and rosiglitazone appear to activate the PEMT pathway to facilitate PC biosynthesis, again considered beneficial for lipoprotein formation and lipid transportation (Lamaziere and Wolf, 2010; Weidner et al., 2012; Ahmadian et al., 2013). Based on the modulation of lipids and pathway analysis, we believe KB also regulates transcription factors (e.g., PPAR-γ), like rosiglitazone, which is already known to regulate genes that influence lipid metabolism. The difference, however, between KB and rosiglitazone may be that KB is more selective and less potent, because the number of lipid species being influenced and the magnitude of fold changes in lipid levels was less than those demonstrated by rosiglitazone, a full PPAR-γ agonist. Similar partial PPAR-γ agonists were found in edible plant herb licorice (*Glycyrrhiza foetida*) (Weidner et al., 2012). To confirm the influence of KB on transcription factors, we plan to perform RNA-Seq to verify the transcription factors regulated by KB corresponding to the lipid profile changes and compare with results from rosiglitazone.

Fatty acid oxidization (FAO) is an important biological process for energy supply and lipid homeostasis. Acylcarnitines are required to shuttle fatty acids through the mitochondrial membrane; thus acylcarnitines, especially long chain acylcarnitines, can be used to reflect the loading of fatty acid flux into mitochondria (Schooneman et al., 2012). In addition to FAO, degradation of ketone bodies (such as acetoacetate, beta-hydroxybutyrate, and acetone) and amino acids (such as valine, leucine, and tryptophan) for energy can also yield short-chain acylcarnitines (such as C3-, C4-, and C-5 acylcarnitines) (Schooneman et al., 2012). In our study, the vehicle-db/db mice had overall lower acylcarnitine levels than vehicle-WT mice in fasting status, indicating that db/db mice were less capable of utilizing either fatty acids or amino acids to adapt to starvation (Muoio, 2014). The PPAR-γ agonist rosiglitazone, reported to be a FAO stimulator (Schooneman et al., 2012; Ahmadian et al., 2013), significantly enhanced long-chain acylcarnitines in the db/db mice, indicating a higher loading of fatty acids into mitochondria to yield energy. Similar increases were observed in the KB-db/db mice. It is generally recognized in humans that the level of FAO can be reflected by the level of medium-chain acylcarnitines (Lehmann et al., 2010; Zhang et al., 2017). However, in the current study, except for C14 acylcarnitines, most of the other medium-chain species were too low to achieve validated quantitation using the Biocrates p180 assay. Nevertheless, we believe our data supports the suggestion that KB accelerates the transformation of fatty acids to acylcarnitines; but the actual FAO level needs to be confirmed using assays that evaluate citric acid cycle oxidation and the respiratory quotient (Speakman, 2013). Unlike KB and rosiglitazone, metformin treatment increased long chain acylcarnitine (*e.g*., C16-OH), but reduced short-chain acylcarnitines, corresponding to its anti-gluconeogenesis effect via reducing burning of amino acids for energy metabolism (Takashima et al., 2010; Viollet et al., 2012). Since KB showed almost no influence on amino acids and biogenic amines (Table S3), perturbation of these compounds by metformin and rosiglitazone, as well the biochemistry outcomes are not further discussed in this paper.

Chronic inflammation and metabolic dysfunction interact to serve as both cause and consequence for diabetes (Esser et al., 2014). This crosstalk was clearly demonstrated in our study, as the diabetic model presented strikingly higher overall cytokine levels compared to WT mice (Figure 4). KB treatment resulted in notable anti-inflammatory effects, reducing the level of 29 of 80 measured cytokines (Table 3), including interleukins, chemokines, insulin- like growth factors, and other growth factors such as HGF and GDNF. This response is not only considered to alleviate insulin resistance and diminish metabolic disorder, which are aggravated by unchecked inflammatory responses (Esser et al., 2014), but also to be beneficial for reducing risk of diabetic complications associated with macro- and micro-angiopathy (Mitamura et al., 2005; Clermont et al., 2006; Konya, 2014). The PPAR-γ agonist has been shown to interfere with immune-related genes to block inflammatory responses (Ahmadian et al., 2013), and coincident with our results rosiglitazone reduced all inflammatory markers in db/db mice after treatment. For some cytokines, the reduction caused by rosiglitazone-db/db vs. vehicle-db/db was even greater than the fold change between vehicle-WT and vehicle-db/db. This aggressive down-regulation may indicate a potential risk of immune compromise during long-term usage, although limited attention has been paid to this possible side effect. Although very effective at maintaining glucose homeostasis, compared to KB and rosiglitazone, the investigated lower-range dose of metformin exhibited the least anti-inflammatory responses, especially for down-regulating interleukins.

Pathway enrichment analysis (GeneGo MetaCore™) was used to evaluate which endogenous pathways were influenced by KB (Table S4) and networks can be seen in Figure 5. Based on the relevant enriched pathways, KB-influenced pathways were primarily related to (1) inflammatory response; (2) positive regulation of metabolic process; (3) lipoprotein assembly, localization, transportation, and clearance; and (4) positive response of peptide hormone. According to the analysis criteria of selected network objects in Figure 5, we believe that KB regulates nuclear transcription factors which inhibit down-stream cytokines and the G-alpha (i)-specific peptide G-protein-coupled receptors (Gi-GPCRs), modulating insulin signaling and metabolic homeostasis (Berger et al., 2015; Oh and Olefsky, 2016). In addition, interference with nuclear transcription factors also suggests regulation of genes and proteins (such as apolipoproteins) (Yue et al., 2008) for lipid metabolism, substantiated by changes of lipid profiles shown in our lipidomics study.

In summary, from a holistic view, we demonstrated KB is effective in alleviating T2DM symptoms, and the primary mode of action may be through modulation of nuclear transcription factors (such as NF-κB and, or PPAR) to alter lipid metabolism and reduce chronic inflammation, thus eventually facilitating metabolic homeostasis. Although rosiglitazone was excellent at lowering blood glucose, the demonstrated overall disruption of lipid metabolism and the immune system may contribute to weight gain, as well as add risks in liver and cardiovascular outcomes, which have been reported in human subjects (Ahmadian et al., 2013). Since KB and rosiglitazone may impact similar pathways in regulating lipid metabolism and inflammation, increasing dosage and/or long-term exposure of KB may result in adverse effects like those of rosiglitazone. To test this, further evaluation on acute and long-term toxicities is required. With the investigated low dose, metformin appeared to be less effective at regulating lipid metabolism, fatty oxidation, and anti-inflammatory markers compared to either KB or rosiglitazone, although it significantly inhibited fasting blood glucose increase in diabetic mice. This indicated that metformin might regulate lipid metabolism and chronic inflammation requiring a higher dose-range and through another pathway independent to the route for simply influencing glucose metabolism. Our study presents great potential to further develop KB as a nutraceutical using independently, or in combination with low-dose first-line pharmaceuticals, for T2DM prevention and management. Our comparison study with metformin and rosiglitazone provided more information on how these currently used medications may act beyond lowering blood glucose.

## Author Contributions

Y-YL: study design, animal sample collection. LC-MS: based metabolomics, data analysis, and manuscript writing. DS: cytokine array experiment design, execution, analysis and data interpretation. X-MY: animal study design consultant and execution. L-HY: animal study execution and animal sample collection. WP: SIMCA 14 multivariate data analysis. SM: statistical analysis. TF: data interpretation. H-YC: supervisor of animal study, including study design, data collection, analysis. SS: supervisor of metabolomics study and manuscript writing.

### Conflict of Interest Statement

The authors declare that the research was conducted in the absence of any commercial or financial relationships that could be construed as a potential conflict of interest.

## Supplementary Material

Data Sheet 1 Biocrates p180 kit Concentration Data (μM).

## Abbreviations

T2DM: Type 2 diabetes mellitus
LyC: Lycii Cortex
KB: Kukoamine B
TG: Triglycerides
CHEL: Cholesterols
PE: Phosphatidylethanolamine
PI: Phosphatidylinositol
PC: Phosphatidylcholine
PCA: Principal component analysis
TCM: Traditional Chinese Medicine.
