# Supplementary material for: A Metabolomics Approach to Investigate Kukoamine B—A Potent Natural Product With Anti-diabetic Properties
Source: Front Pharmacol. 2019 Jan 22;9:1575. doi: 10.3389/fphar.2018.01575 (PMC6350459; doi:10.3389/fphar.2018.01575)
Supplement: Supplementary file 1 [file Presentation_1.PDF]

A metabolomics approach to investigate kukoamine B - a potent natural product with anti-diabetic properties

Yuan-Yuan Li,[1] Delisha A. Stewart,[1] Xiao-Min Ye,[2] Li-Hua Yin,[2] Wimal W. Pathmasiri,[1] Susan L. McRitchie,[1] Timothy R. Fennell,[3] Hon-Yeung Cheung,[4*] and Susan J. Sumner[1*]

1. NIH Eastern Regional Comprehensive Metabolomics Resource Core, Nutrition Research Institute, Department of Nutrition, University of North Carolina at Chapel Hill, Kannapolis, NC 28081, USA

2. Department of pharmacology, Wuhan Institute for Drug and Medical Device Control, Wuhan, Hubei Province, China

3. Analytical Chemistry and Pharmaceutics, RTI International, Research Triangle Park, NC 27709, USA

4. Department of Biomedical Science, City University of Hong Kong, Tat Chee Avenue, Kowloon, Hong Kong SAR

**Co-Corresponding authors*

Metabolomics: Susan J. Sumner, Email: Susan_Sumner@unc.edu, Phone: +1 (704) 2505067

Animal Study: Hon-Yeung Cheung, Email: bhhonyun@cityu.edu.hk, Phone: +852 34427746

Figure S1 Unsupervised multivariate analysis (PCA) of broad spectrum lipidomics

Lipidomics: negative mode, PCA

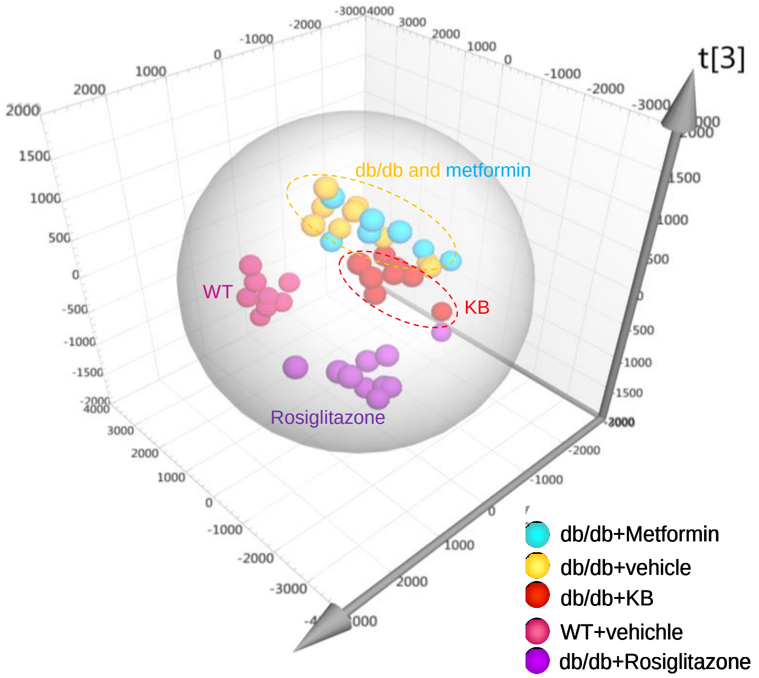

Figure S1 Score scatter plot of the principal component analysis (PCA) for KB-, metformin-, rosiglitazone-, and vehicle-treated db/db mice, and vehicle-treated WT mice. Data was acquired in **negative mode**, R2X (cum) = 0.877.

PCA plots for data acquired in positive mode are shown as Fig 2A.

2. Figure S2 Supervised pairwise comparison (OPLS-DA) between drug-treated group (i.e., KB-, metformin-, and rosiglitazone-db/db) and control (vehicle-db/db) via data acquired by untargeted lipidomics.

Lipidomics: Metformin Vs control (positive mode)

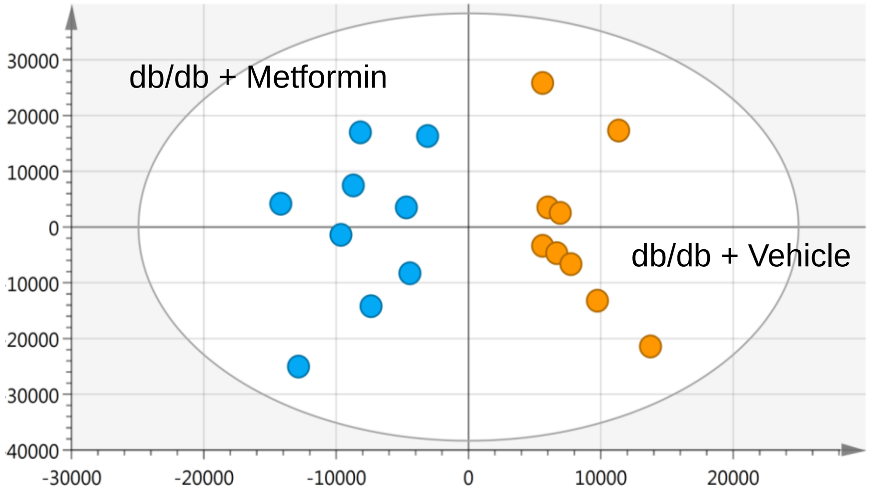

Figure S2A OPLS-DA of metformin-treated db/db mice versus control (Vehicle).R2X (cum)=0.855; R2Y (cum)=0.87; Q2 (cum)= 0.01.

Lipidomics: Rosiglitazone Vs control (positive mode)

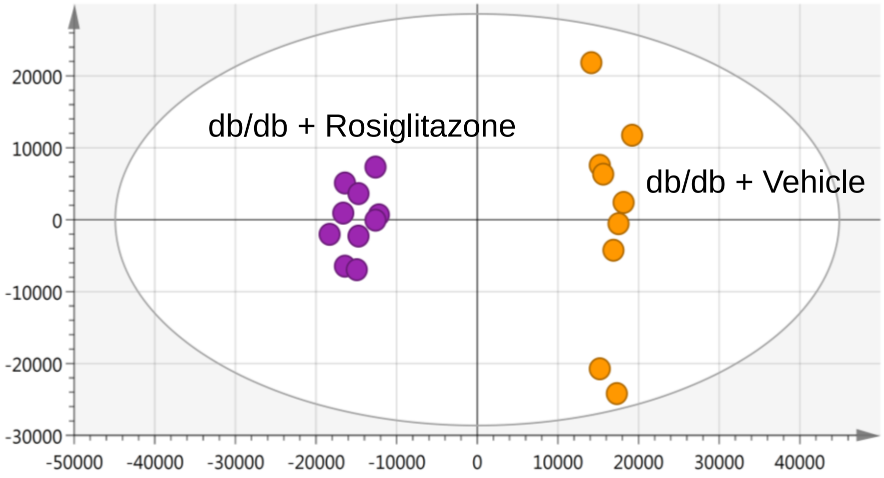

Figure S2B OPLS-DA of rosiglitazone-treated db/db mice versus control. R2X (cum)=0.846; R2Y (cum)=0.988; Q2 (cum)= 0.966.

Lipidomics: KB Vs control (Negative mode)

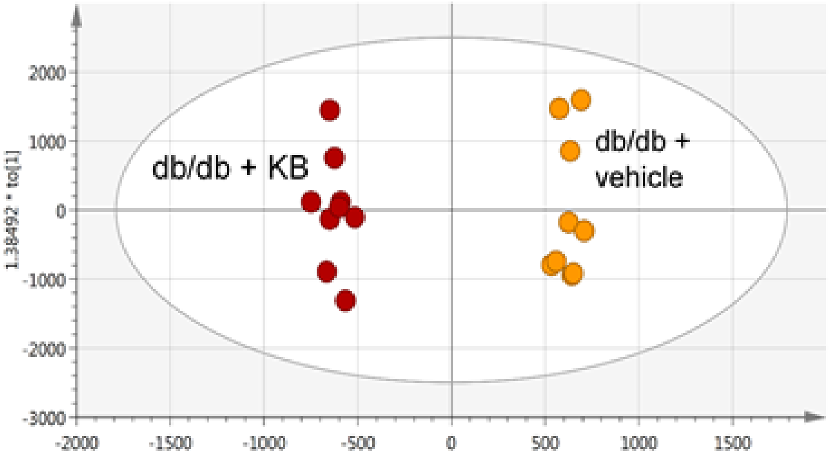

Figure S2C OPLS-DA of KB-treated db/db mice versus control.
R2X (cum)=0.734; R2Y (cum)=0.991; Q2(cum)= 0649.

Lipidomics: Metformin Vs control (Negative mode)

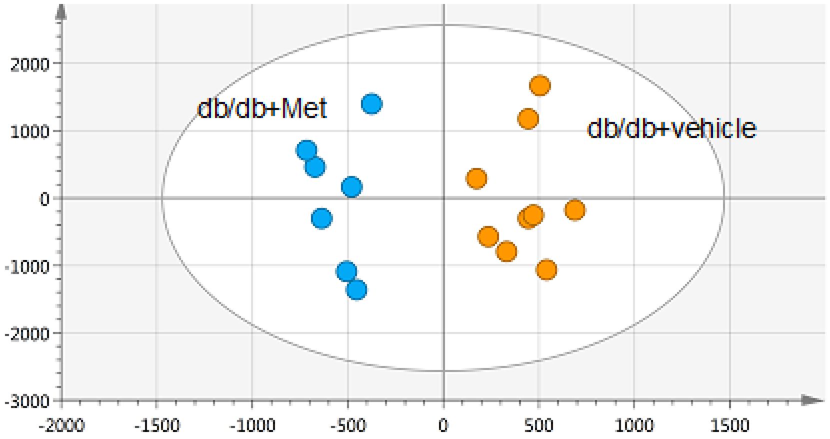

Figure S2D OPLS-DA of metformin-treated db/db mice versus control.
R2X(cum)=0.732; R2Y(cum)=0.926; Q2(cum)= 0.374.

Lipidomics: Rosiglitazone Vs control (Negative mode)

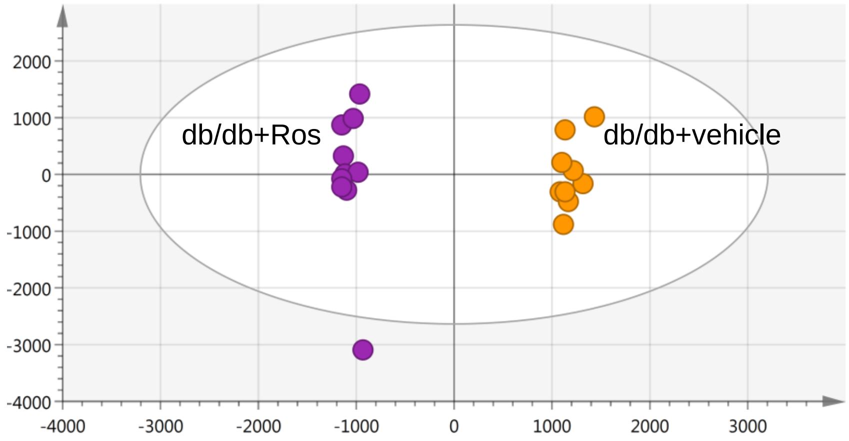

Figure S2E OPLS-DA for rosiglitazone treated db/db mice versus negative control.
R2X(cum)= 0.777; R2Y(cum)=0.993; Q2 (cum)=0.921.

3. Figure S3 Unsupervised multivariate analysis (PCA) of lipid profiles acquired by targeted metabolomics approach (Biocrates p180)

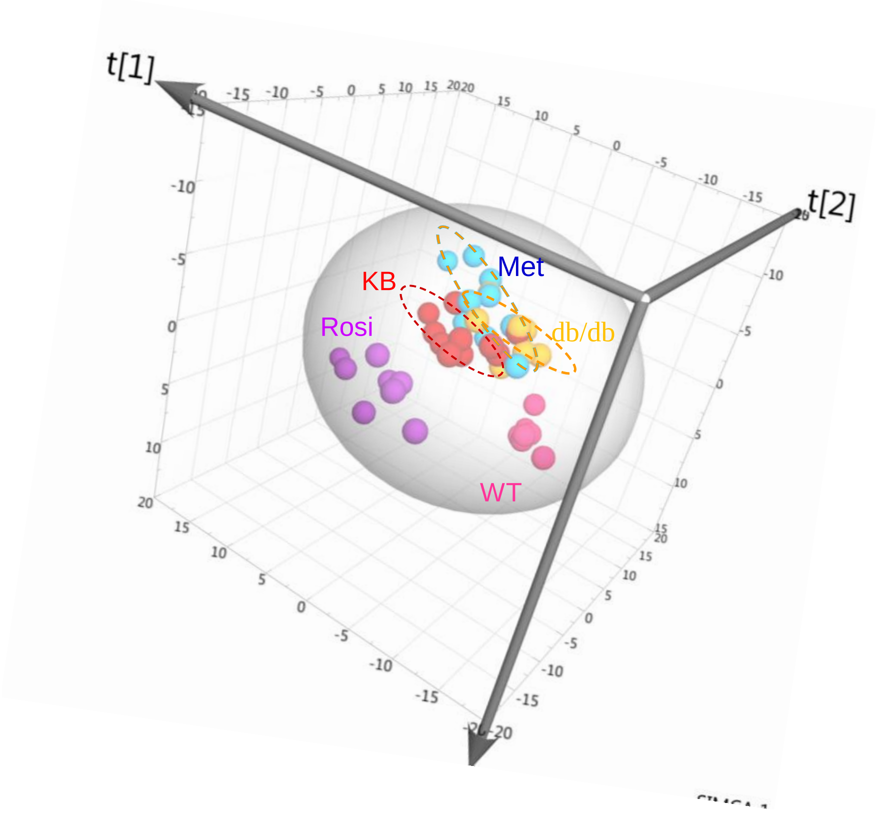

Figure S3 Score scatter plot of the principal component analysis (PCA) for KB-, metformin-, rosiglitazone-, and vehicle-treated db/db mice, and vehicle-treated WT mice. Data was acquired by targeted metabolomics approach via Biocrates p180 kit, R2X (cum) = 0.814.

4. Figure S4 Supervised pairwise multivariate analysis (OPLS-DA) between drug-treated group (i.e., KB-, metformin-, and rosiglitazone- db/db mice) and control (vehicle-db/db) via lipid data acquired by targeted metabolomics (Biocrates p180)

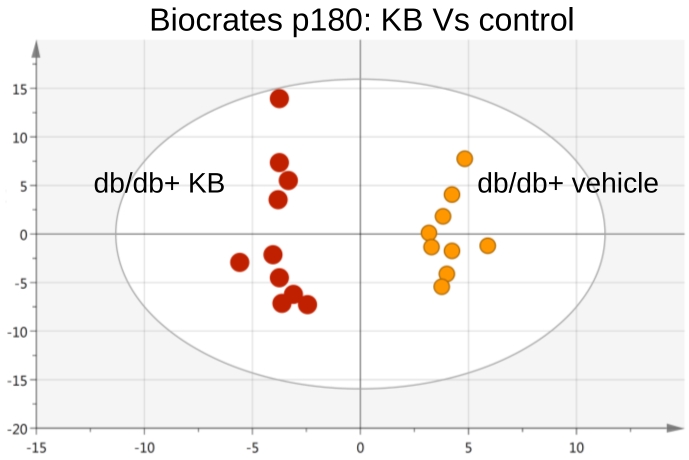

Figure S4A OPLS-DA of KB-treated db/db mice versus control (Lipid acquired by Biocrates p180.
R2X (cum)=0.702; R2Y (cum)=0.964; Q2 (cum)= 0.575

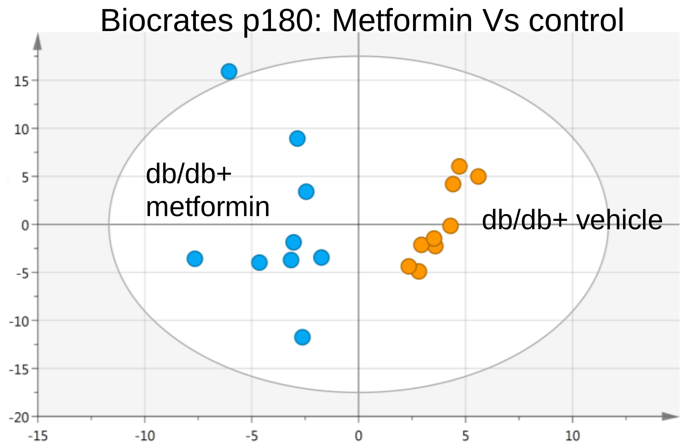

Figure S4B OPLS-DA of metformin-treated db/db mice versus control (Lipid acquired by Biocrates p180.
R2X (cum)=0.69; R2Y (cum)=0.871; Q2 (cum)= 0.237

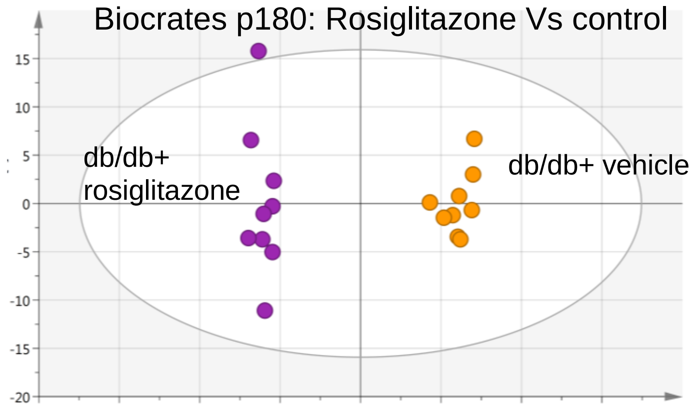

Figure S4C OPLS-DA of rosiglitazone-treated db/db mice versus control (Lipid acquired by Biocrates p180.
R2X (cum)=0.845; R2Y (cum)=0.987; Q2 (cum)= 0.953

Table S1. Serum lipid profiles following metformin treatment in db/db mice.

Accepted Description	Detection Methodology	Lipid Class	Metformin Vs db/db		
			VIP*	*p*-value**	FC***
PC(0:0/20:4(5Z,8Z,11Z,14Z))	pos	LPC	5.7	0.016	1.5
PC(20:4(5Z,8Z,11Z,14Z)/0:0)	neg	LPC	6.4	0.04	1.4
lysoPC a C20:4	Biocrates	LPC	1.4	0.00	1.6
lysoPC a C24:0	Biocrates	LPC	1.2	0.02	1.1
lysoPC a C26:1	Biocrates	LPC	1.4	0.01	1.3
lysoPC a C28:1	Biocrates	LPC	1.3	0.05	1.3
PC(22:6(4Z,7Z,10Z,13Z,16Z,19Z)/16:1(9Z))	pos	PCaa	1.9	0.005	1.9
PC(17:0/20:4(5Z,8Z,11Z,14Z))	neg	PCaa	1.7	0.021	1.3
PC(18:0/20:4(8Z,10Z,12Z,14Z))	neg	PCaa	14.6	0.017	1.3
PC aa C34:1	Biocrates	PCaa	1.3	0.03	1.4
PC aa C36:4	Biocrates	PCaa	1.4	0.03	1.5
PC aa C38:4	Biocrates	PCaa	1.5	0.02	1.5
PC aa C38:5	Biocrates	PCaa	1.3	0.03	1.4
PC aa C38:6	Biocrates	PCaa	1.4	0.02	1.4
PC aa C40:4	Biocrates	PCaa	1.2	0.02	1.4
PC aa C40:5	Biocrates	PCaa	1.1	0.04	1.4
PC aa C40:6	Biocrates	PCaa	1.4	0.02	1.4
PC ae C38:0	Biocrates	PCae	1.3	0.04	1.4
PC ae C40:1	Biocrates	PCae	1.2	0.03	1.2
PC ae C40:3	Biocrates	PCae	1.1	0.04	1.2
PC ae C42:1	Biocrates	PCae	1.2	0.01	1.4
PC ae C42:3	Biocrates	PCae	1.2	0.05	1.3
PE(P-16:0/22:6(4Z,7Z,10Z,13Z,16Z,19Z))	neg	PE	3.4	0.046	-1.3
PE(P-20:0/22:6(4Z,7Z,10Z,13Z,16Z,19Z))	neg	PE	1.5	0.015	-1.3
PG(O-18:0/21:0)	neg	PG	1.1	0.001	2.3
PI(18:0/18:2(9Z,12Z))	neg	PI	3.1	0.017	-1.4
SM(d17:1/24:0)	pos	SM	1.1	0.046	-1.1
SM(d18:1/18:0)	pos	SM	2.4	0.026	1.4
SM(d18:1/18:1(9Z))	pos	SM	1.3	0.045	1.2
SM(d16:1/20:0)	neg	SM	3.1	0.016	1.3
SM(d18:1/15:0)	neg	SM	1.4	0.01	1.3
SM (OH) C14:1	Biocrates	SM	1.4	0.01	1.3
SM C18:0	Biocrates	SM	1.4	0.02	1.4
SM C18:1	Biocrates	SM	1.4	0.02	1.3
SM C20:2	Biocrates	SM	1.3	0.01	1.5
1-(Octadecyloxy)-3-(palmitoyloxy)-2-propanyl (9Z,12Z,15Z)-9,12,15-octadecatrienoate	pos	TG	1	0.022	-1.7
1-(Octadecyloxy)-3-(stearoyloxy)-2-propanyl (6Z,9Z,12Z)-6,9,12-octadecatrienoate	pos	TG	1.4	0.01	-1.8
1-[(9Z)-9-Octadecenoyloxy]-3-(stearoyloxy)-2-propanyl (9Z,12Z)-9,12-nonadecadienoate	pos	TG	3.5	0.027	-1.9

2-[(11Z)-11-Icosenoyloxy]-3-(nonadecanoyloxy)propyl henicosanoate	pos	TG	1.2	0.015	-2.2
2-[(11Z)-11-Octadecenoyloxy]-3-(octadecyloxy)propyl (5Z,8Z,11Z)-5,8,11-icosatrienoate	pos	TG	1.1	0.017	-1.6
2-[(5Z,8Z,11Z,14Z,17Z)-5,8,11,14,17-	pos	TG	1.2	0.009	-1.9
2-[(9Z)-9-Hexadecenoyloxy]-3-(palmitoyloxy)propyl (5Z,8Z,11Z)-5,8,11-icosatrienoate	pos	TG	3.2	0.029	-1.4
3-(Heptadecanoyloxy)-2-[(9Z,12Z)-9,12-octadecadienoyloxy]propyl icosanoate	pos	TG	2.4	0.017	-2.4
3-[(11Z)-11-Icosenoyloxy]-2-(octadecyloxy)propyl (5Z,8Z,11Z,14Z,17Z)-5,8,11,14,17-icosapentaenoate	pos	TG	1.4	0.031	-1.5
TG(14:1(9Z)/22:1(13Z)/o-18:0)	pos	TG	2.7	0.009	-1.9
TG(15:0/20:2n6/o-18:0)	pos	TG	2.3	0.007	-1.8
TG(16:0/16:1(9Z)/o-18:0)	pos	TG	1.5	0.008	-1.7
TG(17:1(9Z)/20:0/21:0)[iso6]	pos	TG	1.9	0.014	-2.1
TG(18:0/20:1(11Z)/20:2(11Z,14Z))[iso6]	pos	TG	4	0.008	-2.3
TG(18:0/o-18:0/20:3n6)	pos	TG	1.8	0.007	-2
TG(18:1(9Z)/19:0/22:2(13Z,16Z))[iso6]	pos	TG	4.4	0.024	-1.7
TG(18:1(9Z)/19:1(9Z)/20:1(11Z))[iso6]	pos	TG	6.6	0.04	-1.6
TG(18:1(9Z)/20:1(11Z)/o-18:0)	pos	TG	2.4	0.011	-1.9
TG(18:1/21:0/22:0)	pos	TG	1.3	0.015	-2.2
TG(18:2(9Z,12Z)/20:2(11Z,14Z)/22:0)[iso6]	pos	TG	3.1	0.01	-2.8
TG(22:6(4Z,7Z,10Z,13Z,16Z,19Z)/20:1(11Z)/o-18:0)	pos	TG	8.1	0.027	-1.6

Detection of lipids by broad spectrum lipidomics was performed in positive and negative modes; Biocrates p180 kit was used for targeted quantitation of lipids. Lipid classes detected include: LPC, lysophosphatidylcholine; LPE, lysophosphatidylethanolamine; MG, monoglyceride; PC aa, diacyl-phosphatidylcholine; PC ae, alkylacyl-phosphatidylcholine; PE, phosphatidylethanolamine; PI, phosphatidylinositol; PS, phosphatidylserine; SM, sphingomyelin; TG, triglyceride. VIP*, variable influence on projection; FC***, fold change (compared to "vehicle-db/db" as reference based on mean); p-value**, significance examination via *t*-test. Cut-off criteria for significance of lipids responding to treatment: VIP≥1.0 and $p<0.05$.

Table S2. Serum lipid profiles following treatment with rosiglitazone in db/db mice.

Accepted Description	Detection Methodology	Lipid Class	Rosiglit Vs db/db		
			VIP*	p-value**	FC***
Cer(d18:1/24:1(15Z))	pos	Cer	1.4	<.001	2.8
(2S)-3-Hydroxy-2-(stearoyloxy)propyl (8Z,11Z,14Z)-8,11,14-icosatrienoate	pos	DG	1.3	<.001	3.9
DG(18:0/20:4(5Z,8Z,11Z,14Z)/0:0)[iso2]	pos	DG	3.3	<.001	1.5
DHA	neg	FFA	1.1	0.01	-1.8
(2R)-2-Hydroxy-3-(palmitoyloxy)propyl 2-(trimethylammonio)ethyl phosphate	pos	LPC	5.3	0.047	1.3
PC(0:0/18:1(9E))	pos	LPC	6.9	<.001	2.8
PC(0:0/20:4(5Z,8Z,11Z,14Z))	pos	LPC	3.6	0.034	1.5
PC(16:0/16:0)	pos	LPC	3.8	<.001	1.6
PC(16:1(9Z)/0:0)	pos	LPC	2.4	<.001	2.8
PC(20:3(8Z,11Z,14Z)/0:0)	pos	LPC	3.7	0.004	2.7
PC(15:0/0:0)	neg	LPC	4.0	0.04	1.2
PC(15:1(9Z)/0:0)	neg	LPC	1.6	<.0001	3.2
PC(16:0/0:0)[rac]	neg	LPC	5.3	0.04	1.2
PC(16:1(9E)/0:0)	neg	LPC	2.2	<.0001	3.2
PC(17:1(10Z)/0:0)	neg	LPC	4.5	<.0001	2.7
PC(18:1(6Z)/0:0)	neg	LPC	7.5	<.0001	2.7
PC(20:1(11Z)/0:0)	neg	LPC	1.5	<.0001	3.3
PC(20:4(5Z,8Z,11Z,14Z)/0:0)	neg	LPC	4.7	0.005	1.7
lysoPC a C16:0	Biocrates	LPC	1.0	0.02	1.3
lysoPC a C16:1	Biocrates	LPC	1.3	0.00	3.0
lysoPC a C17:0	Biocrates	LPC	1.2	0.00	-1.7
lysoPC a C18:1	Biocrates	LPC	1.3	0.00	2.7
lysoPC a C20:3	Biocrates	LPC	1.2	0.00	2.8
lysoPC a C20:4	Biocrates	LPC	1.0	0.00	1.7
lysoPC a C26:0	Biocrates	LPC	1.2	0.00	2.2
lysoPC a C26:1	Biocrates	LPC	1.0	0.01	1.4

lysoPC a C28:0	Biocrates	LPC	1.1	0.00	1.4
lysoPC a C28:1	Biocrates	LPC	1.1	0.00	1.5
PC(16:0/18:1(9Z))	pos	Pcaa	24	<.001	2.4
PC(16:1(9Z)/16:0)	pos	PCaa	6.2	<.001	4.4
PC(17:1(9Z)/16:0)	pos	PCaa	1.8	<.001	2.2
PC(18:0/18:1(16Z))	pos	PCaa	16.8	<.001	1.5
PC(18:0/22:4(7Z,10Z,13Z,16Z))	pos	PCaa	1.3	<.001	1.7
PC(18:1(9Z)/17:0)	pos	PCaa	1.5	<.001	1.4
PC(18:1(9Z)/18:0)	pos	PCaa	13.3	<.001	2.7
PC(18:2(9Z,12Z)/14:0)	pos	PCaa	1.2	<.001	2.3
PC(18:2(9Z,12Z)/17:0)	pos	PCaa	1	<.001	1.6
PC(18:2(9Z,12Z)/19:0)	pos	PCaa	1.9	0.01	-1.5
PC(18:2(9Z,12Z)/20:0)	pos	PCaa	4.1	<.001	2.9
PC(20:3(5Z,8Z,11Z)/18:0)	pos	PCaa	16.1	<.001	2.2
PC(20:3(5Z,8Z,11Z)/20:1(11Z))	pos	PCaa	3	<.001	7
PC(20:4(8E,11E,14E,17E)/16:0)	pos	PCaa	20.3	<.001	1.7
PC(20:4(8Z,11Z,14Z,17Z)/20:0)	pos	PCaa	1	<.001	1.8
PC(22:6(4Z,7Z,10Z,13Z,16Z,19Z)/16:1(9Z))	pos	PCaa	1.7	<.001	1.7
PC(22:6(4Z,7Z,10Z,13Z,16Z,19Z)/17:0)	pos	PCaa	2.1	<.001	-1.8
PC(22:6(4Z,7Z,10Z,13Z,16Z,19Z)/18:0)	pos	PCaa	2.3	<.001	-2.3
PC(15:0/20:4(5Z,8Z,11Z,14Z))	neg	PCaa	1.6	0.00	1.7
PC(15:0/22:6(4Z,7Z,10Z,13Z,16Z,19Z))	neg	PCaa	1.3	0.01	1.3
PC(16:0/20:4(5E,8E,11E,14E))	neg	PCaa	10.6	0.00	1.6
PC(17:0/15:0)	neg	PCaa	3.5	0.00	1.6
PC(17:0/19:1(9Z))	neg	PCaa	10.0	<.0001	2.6
PC(17:0/22:6(4Z,7Z,10Z,13Z,16Z,19Z))	neg	PCaa	1.5	<.0001	-2.0
PC(18:0/20:3(5Z,11Z,14Z))	neg	PCaa	10.9	<.0001	2.1
PC(18:0/20:4(5Z,8Z,11Z,14Z))	neg	PCaa	9.4	<.0001	4.3

PC(18:0/22:5(4Z,7Z,10Z,13Z,16Z))	neg	PCaa	1.5	0.02	1.2
PC(18:0/22:6(4Z,7Z,10Z,13Z,16Z,19Z))	neg	PCaa	6.8	<.0001	-1.4
PC(18:1(11Z)/18:3(6Z,9Z,12Z))	neg	PCaa	3.1	0.001	-1.4
PC(18:1(9Z)/22:6(4Z,7Z,10Z,13Z,16Z,19Z))	neg	PCaa	5.6	<.0001	1.8
PC(19:0/18:2(9Z,12Z))	neg	PCaa	1.2	0.013	-1.3
PC(20:1(11Z)/20:3(8Z,11Z,14Z))	neg	PCaa	2.0	<.0001	6.1
PC(21:0/14:1(9Z))	neg	PCaa	1.1	0.002	1.3
PC(22:2(13Z,16Z)/14:1(9Z))	neg	PCaa	10.8	<.0001	1.8
PC(24:1(15Z)/14:1(9Z))	neg	PCaa	3.1	<.0001	2.6
PC(P-18:0/16:0)	neg	PCaa	1.3	0.000	1.7
PC aa C28:1	Biocrates	PCaa	1.3	0.00	1.7
PC aa C30:0	Biocrates	PCaa	1.2	0.00	1.7
PC aa C32:0	Biocrates	PCaa	1.1	0.00	1.4
PC aa C32:1	Biocrates	PCaa	1.2	0.00	4.2
PC aa C32:2	Biocrates	PCaa	1.1	0.00	2.4
PC aa C32:3	Biocrates	PCaa	1.1	0.00	1.4
PC aa C34:1	Biocrates	PCaa	1.3	0.00	3.2
PC aa C34:3	Biocrates	PCaa	1.2	0.00	2.1
PC aa C34:4	Biocrates	PCaa	1.1	0.00	1.9
PC aa C36:0	Biocrates	PCaa	1.2	0.00	-1.5
PC aa C36:1	Biocrates	PCaa	1.3	0.00	2.4
PC aa C36:3	Biocrates	PCaa	1.3	0.00	2.5
PC aa C36:4	Biocrates	PCaa	1.1	0.01	1.8
PC aa C36:6	Biocrates	PCaa	1.0	0.03	1.4
PC aa C38:0	Biocrates	PCaa	1.1	0.00	-1.4
PC aa C38:1	Biocrates	PCaa	1.1	0.00	1.6
PC aa C38:3	Biocrates	PCaa	1.3	0.00	2.3
PC aa C38:4	Biocrates	PCaa	1.1	0.00	1.5

PC aa C38:5	Biocrates	PCaa	1.2	0.00	2.1
PC aa C40:3	Biocrates	PCaa	1.3	0.00	2.1
PC aa C40:4	Biocrates	PCaa	1.3	0.00	2.3
PC aa C40:5	Biocrates	PCaa	1.0	0.02	1.3
PC aa C42:1	Biocrates	PCaa	1.0	0.01	-1.3
PC aa C42:6	Biocrates	PCaa	1.0	0.01	1.3
PC(O-16:0/22:6(4Z,7Z,10Z,13Z,16Z,19Z))	pos	PCae	1.3	0.026	-1.3
PC(O-18:0/16:1(9Z))	pos	PCae	2	<.001	1.9
PC(O-16:0/20:4(5Z,8Z,11Z,14Z))	neg	PCae	2.0	0.00	1.5
PC(O-16:0/22:6(4Z,7Z,10Z,13Z,16Z,19Z))	neg	PCae	1.1	0.01	-1.3
PC ae C32:1	Biocrates	PCae	1.0	0.03	1.3
PC ae C34:1	Biocrates	PCae	1.2	0.00	1.8
PC ae C34:2	Biocrates	PCae	1.0	0.01	-1.3
PC ae C34:3	Biocrates	PCae	1.1	0.00	-1.4
PC ae C36:1	Biocrates	PCae	1.0	0.02	1.3
PC ae C36:2	Biocrates	PCae	1.2	0.00	-1.6
PC ae C38:0	Biocrates	PCae	1.0	0.01	1.6
PC ae C38:2	Biocrates	PCae	1.1	0.00	-1.4
PC ae C38:3	Biocrates	PCae	1.0	0.01	1.3
PC ae C38:6	Biocrates	PCae	1.1	0.00	-1.4
PC ae C40:1	Biocrates	PCae	1.1	0.00	-1.4
PC ae C40:2	Biocrates	PCae	1.0	0.01	-1.3
PC ae C40:3	Biocrates	PCae	1.1	0.00	1.3
PC ae C40:5	Biocrates	PCae	1.0	0.04	-1.2
PC ae C40:6	Biocrates	PCae	1.3	0.00	-1.8
PC ae C42:1	Biocrates	PCae	1.0	0.02	1.3
PC ae C42:3	Biocrates	PCae	1.0	0.01	-1.3
PC ae C42:4	Biocrates	PCae	1.1	0.00	-1.4

PC ae C42:5	Biocrates	PCae	1.1	0.00	-1.2
PC ae C44:4	Biocrates	PCae	1.0	0.01	-1.3
PC ae C44:5	Biocrates	PCae	1.3	0.00	-1.6
PC ae C44:6	Biocrates	PCae	1.2	0.00	-1.4
PE(P-18:0/22:6(4Z,7Z,10Z,13Z,16Z,19Z))	pos	PE	1	<.001	-2.1
PE(18:1(9Z)/0:0)	neg	PE	1.2	<.0001	2.5
PE(O-18:0/18:2(9Z,12Z))	neg	PE	1.4	0.000	-2.4
PE(O-18:0/22:4(7Z,10Z,13Z,16Z))	neg	PE	1.0	0.002	-2.1
PE(O-18:0/22:6(4Z,7Z,10Z,13Z,16Z,19Z))	neg	PE	4.3	<.0001	-2.6
PE(O-20:0/18:2(9Z,12Z))	neg	PE	1.1	0.000	-2.3
PE(O-20:0/20:4(5Z,8Z,11Z,14Z))	neg	PE	1.6	0.001	-1.9
PE(O-20:0/22:6(4Z,7Z,10Z,13Z,16Z,19Z))	neg	PE	2.0	<.0001	-2.6
PE(P-16:0/22:6(4Z,7Z,10Z,13Z,16Z,19Z))	neg	PE	2.9	0.000	-1.8
PE(P-18:0/18:2(9Z,12Z))	neg	PE	1.2	0.001	-2.3
PE(P-18:0/20:4(5Z,8Z,11Z,14Z))	neg	PE	2.8	0.001	-1.8
PE(P-18:0/22:4(7Z,10Z,13Z,16Z))	neg	PE	1.5	<.0001	-2.9
PE(P-18:0/22:6(4Z,7Z,10Z,13Z,16Z,19Z))	neg	PE	2.8	<.0001	-2.4
PE(P-20:0/20:4(5Z,8Z,11Z,14Z))	neg	PE	1.2	0.000	-2.0
PE(P-20:0/22:6(4Z,7Z,10Z,13Z,16Z,19Z))	neg	PE	1.6	<.0001	-2.6
PE-NMe2(16:0/18:1(9Z))	neg	PENMe	3.2	<.0001	3.0
PI(18:4(6Z,9Z,12Z,15Z)/20:1(11Z))	pos	PI	1.1	<.001	2.1
PI(18:0/20:4(5Z,8Z,11Z,14Z))	pos	PI	5	0.002	1.4
PI(16:0/18:2(9Z,12Z))	neg	PI	1.2	0.02	-1.4
PI(16:0/20:4(5Z,8Z,11Z,14Z))	neg	PI	2.2	0.00	1.3
PI(18:0/18:2(9Z,12Z))	neg	PI	1.4	0.02	-1.3
PI(18:0/20:3(8Z,11Z,14Z))	neg	PI	4.6	<.0001	2.7
PI(18:0/20:3(8Z,11Z,14Z))	neg	PI	3.8	<.0001	4.4
PI(18:0/20:4(5Z,8Z,11Z,14Z))	neg	PI	8.9	0.00	1.3

PI(18:1(9Z)/18:2(9Z,12Z))	neg	PI	1.6	0.00	1.5
PI(18:1(9Z)/20:4(5Z,8Z,11Z,14Z))	neg	PI	3.9	<.0001	2.4
PS(O-20:0/17:1(9Z))	pos	PS	2.8	0.01	-1.4
PS(19:1(9Z)/22:2(13Z,16Z))	neg	PS	2.1	<.0001	2.0
PS(22:4(7Z,10Z,13Z,16Z)/19:0)	neg	PS	1.5	0.01	1.2
PS(22:4(7Z,10Z,13Z,16Z)/19:1(9Z))	neg	PS	2.5	<.0001	1.5
SM(d17:1/17:0)	pos	SM	6.2	<.001	1.4
SM(d17:1/24:0)	pos	SM	1.6	<.001	-2
SM(d18:1/24:0)	pos	SM	1.8	<.001	-1.7
SM(d18:2/22:0)	pos	SM	1	0.01	-1.3
SM(d18:2/24:0)	pos	SM	6.5	<.001	1.9
SM(d18:1/23:0)	neg	SM	1.6	0.00	-1.6
SM(d18:1/24:0)	neg	SM	2.3	0.00	-1.6
SM(d18:1/24:1(15Z))	neg	SM	6.5	<.0001	1.9
SM (OH) C16:1	Biocrates	SM	1.2	0.00	-1.8
SM (OH) C22:1	Biocrates	SM	1.3	0.00	-1.8
SM (OH) C22:2	Biocrates	SM	1.1	0.00	-1.4
SM C24:1	Biocrates	SM	1.1	0.00	1.7
(2R)-3-[(9Z)-9-Hexadecenoyloxy]-2-[(9Z,12Z)-9,12-octadecadienoyloxy]propyl [illegible]	pos	TG	2.9	0.002	-3.9
(2R)-3-[(9Z,12Z)-9,12-Heptadecadienoyloxy]-2-[(5Z,8Z,11Z,14Z,17Z)-5,8,11,14,17- [illegible]	pos	TG	2.9	<.001	-5.2
1-(Octadecyloxy)-3-(stearoyloxy)-2-propanyl (6Z,9Z,12Z)-6,9,12-octadecatrienoate	pos	TG	1.1	0.001	-2.6
1-[(9Z)-9-Octadecenoyloxy]-3-(stearoyloxy)-2-propanyl (9Z,12Z)-9,12-nonadecadienoate	pos	TG	2.5	0.011	-2.1
2-[(11Z)-11-Octadecenoyloxy]-3-(octadecyloxy)propyl (5Z,8Z,11Z)-5,8,11-	pos	TG	1.0	<.001	-2.8
2-[(5Z,8Z,11Z,14Z,17Z)-5,8,11,14,17-Icosapentaenoyloxy]-3-(octadecyloxy)propyl (13Z)- [illegible]	pos	TG	1.0	<.001	-3.2
2-[(9Z)-9-Hexadecenoyloxy]-3-(palmitoyloxy)propyl (5Z,8Z,11Z)-5,8,11-icosatrienoate	pos	TG	2.8	0.002	-1.8
3-(Heptadecanoyloxy)-2-[(9Z,12Z)-9,12-octadecadienoyloxy]propyl icosanoate	pos	TG	1.7	0.008	-3
3-[(11Z)-11-Icosenoyloxy]-2-(octadecyloxy)propyl (5Z,8Z,11Z,14Z,17Z)-5,8,11,14,17-	pos	TG	1.5	<.001	-2.8
3-[(6Z,9Z,12Z,15Z)-6,9,12,15-Octadecatetraenoyloxy]-2-[(9Z)-9- [illegible]	pos	TG	4.4	0.009	-2.3

TG(14:0/16:1(9Z)/18:2(9Z,12Z))[iso6]	pos	TG	1.1	0.044	-1.7
TG(14:1(9Z)/20:2(11Z,14Z)/22:2(13Z,16Z))[iso6]	pos	TG	1.6	<.001	-4.8
TG(14:1(9Z)/22:1(13Z)/o-18:0)	pos	TG	2.1	0.002	-2.4
TG(15:0/20:2n6/o-18:0)	pos	TG	1.8	0.001	-2.4
TG(16:0/16:1(9Z)/18:1(9Z))[iso6]	pos	TG	7.8	0.047	-1.5
TG(16:0/16:1(9Z)/o-18:0)	pos	TG	1.2	0.001	-2.2
TG(16:0/18:1(9Z)/18:2(9Z,12Z))[iso6]	pos	TG	17.5	0.012	-1.8
TG(16:1(9Z)/16:1(9Z)/20:5(5Z,8Z,11Z,14Z,17Z))[iso3]	pos	TG	1	0.003	-3.6
TG(16:1(9Z)/18:2(9Z,12Z)/18:3(9Z,12Z,15Z))[iso6]	pos	TG	2.6	0.005	-3.8
TG(16:1(9Z)/18:2(9Z,12Z)/20:5(5Z,8Z,11Z,14Z,17Z))[iso6]	pos	TG	2.2	<.001	-4.3
TG(16:1(9Z)/18:2(9Z,12Z)/22:4(7Z,10Z,13Z,16Z))[iso6]	pos	TG	13.2	0.002	-2.7
TG(17:1(9Z)/18:0/18:0)[iso3]	pos	TG	1.7	0.007	-2.4
TG(17:1(9Z)/20:0/21:0)[iso6]	pos	TG	1.2	0.018	-2
TG(18:0/18:3(9Z,12Z,15Z)/22:6(4Z,7Z,10Z,13Z,16Z,19Z))[iso6]	pos	TG	9.9	<.001	-3.8
TG(18:0/o-18:0/20:3n6)	pos	TG	1.4	0.001	-2.7
TG(18:1(9Z)/19:1(9Z)/20:1(11Z))[iso6]	pos	TG	5.1	0.008	-2
TG(18:1(9Z)/20:1(11Z)/o-18:0)	pos	TG	1.5	0.02	-1.7
TG(18:2(9Z,12Z)/20:2(11Z,14Z)/22:0)[iso6]	pos	TG	1.8	0.033	-2.1
TG(18:2(9Z,12Z)/20:5(5Z,8Z,11Z,14Z,17Z)/20:5(5Z,8Z,11Z,14Z,17Z))[iso3]	pos	TG	1	<.001	-7.8
TG(18:3(6Z,9Z,12Z)/18:4(6Z,9Z,12Z,15Z)/20:3(8Z,11Z,14Z))[iso6]	pos	TG	6.2	0.002	-5.2
TG(18:4(6Z,9Z,12Z,15Z)/20:1(11Z)/22:4(7Z,10Z,13Z,16Z))[iso6]	pos	TG	4.7	<.001	-2.6
TG(20:1(11Z)/20:5(5Z,8Z,11Z,14Z,17Z)/22:6(4Z,7Z,10Z,13Z,16Z,19Z))[iso6]	pos	TG	3.9	<.001	-3.2
TG(20:2(11Z,14Z)/22:6(4Z,7Z,10Z,13Z,16Z,19Z)/22:6(4Z,7Z,10Z,13Z,16Z,19Z))[iso3]	pos	TG	2.6	<.001	-4.4
TG(20:4(5Z,8Z,11Z,14Z)/22:6(4Z,7Z,10Z,13Z,16Z,19Z)/22:6(4Z,7Z,10Z,13Z,16Z,19Z))[iso3]	pos	TG	1	<.001	-6
20:4 Cholesteryl ester	pos	CHO	7.2	0.018	1.2

Detection of lipids by broad spectrum lipidomics was performed in positive and negative modes; Biocrates p180 kit was used for targeted quantitation of lipids. . Lipid classes detected include: LPC, lysophosphatidylcholine; LPE, lysophosphatidylethanolamine; MG, monoglyceride; PC aa, diacyl-phosphatidylcholine; PC ae, alkylacyl-phosphatidylcholine; PE, phosphatidylethanolamine; PI, phosphatidylinositol; PS, phosphatidylserine; SM, sphingomyelin; TG, triglyceride. CHO, cholesterol. VIP*, variable influence on projection; FC***, fold change

(compared to “vehicle-db/db” as reference based on mean); p-value**, significance examination via *t*-test. Cut-off criteria for significance of lipids responding to treatment: VIP≥1.0 and $p<0.05$.

Table S3 Biogenic amine and amino acid profiles following treatment with KB in db/db mice.

Name in Biocrates	Full name	p-value*	FC**
Ala	Alanine	-1.19	0.07
Arg	Arginine	-1.07	0.68
Asn	Asparagine	1.12	0.16
Asp	Aspartate	-1.03	0.82
Cit	Citrulline	-1.25	0.39
Gln	Glutamine	1.14	0.01
Glu	Glutamate	1.11	0.36
Gly	Glycine	1.04	0.52
His	Histidine	1.12	0.13
Ile	Isoleucine	1.00	0.94
Leu	Leucine	1.04	0.62
Lys	Lysine	-1.09	0.28
Met	Methionine	1.01	0.92
Orn	Ornithine	-1.04	0.80
Phe	Phenylalanine	-1.02	0.82
Pro	Proline	1.00	0.98
Ser	Serine	1.03	0.75
Thr	Threonine	1.01	0.95
Trp	Tryptophan	-1.09	0.49
Tyr	Tyrosine	-1.06	0.51
Val	Valine	-1.03	0.66
Ac-Orn	Ac-Orn	-1.10	0.35
ADMA	ADMA	-1.11	0.14
alpha-AAA	alpha-AAA	1.02	0.86
Carnosine	Carnosine	-1.29	0.06
Creatinine	Creatinine	1.13	0.12
Histamine	Histamine	-1.14	0.52
Kynurenine	Kynurenine	-1.07	0.57
Met-SO	Met-SO	-1.04	0.81
Putrescine	Putrescine	1.47	0.01
Serotonin	Serotonin	-1.05	0.47
Spermidine	Spermidine	1.22	0.15
Spermine	Spermine	1.00	0.97
t4-OH-Pro	t4-OH-Pro	-1.01	0.88
Taurine	Taurine	-1.01	0.50
total DMA	total DMA	1.00	0.99

Note: Analysis performed using Biocrates p180 kit. Analytes measuring "lower than detection limit" or not passing QC were excluded. If analyte had a "response to KB", the analyte significantly changed in KB-treated db/db mice versus vehicle-treated db/db mice (control). Fold change (FC) uses "vehicle-db/db" as reference based on mean. The -p-value was calculated by *t*-test.-. Threshold criteria for significance: *FC>1.5, or **p<0.05.

Table S3. Cytokine profiles following treatment with metformin in db/db mice.

Cytokines	Full name	Fold Change**	p-value*
MIP-3 alpha	CCL19	-2	0.643
EGF	Epidermal growth factor	-1.8	0.386
Fractalkine	CX3CL1	-1.8	0.505
MCP-4	CCL13, monocyte chemoattractant protein 4	-1.8	0.877
GCP-2	Granulocyte chemotactic protein 2, or CXCL6	-1.7	0.386
IGFBP-2	Insulin like growth factor binding protein 2	-1.7	0.572
MIP-1delta	macrophage inflammatory protein, CCL3 AND CLL4	-1.7	0.443
HGF	Heptocyte growth factor	-1.6	0.572
IGFBP-4	Insulin like growth factor binding protein 4	-1.6	0.959
Leptin	Leptin	-1.6	0.386
MCP-2		-1.6	0.572
MIF	Macrophage migration inhibitory factor	-1.6	0.505
NT-4	neorotrophin-4	-1.6	0.877
Osteoprotegerin	tumor necrosis factor receptor superfamily member 11B	-1.6	0.718
GDNF	Glial cell line-derived neurotrophic factor	-1.5	0.246

Note: Inflammatory cytokine arrays (RayBiotech) were used to detect changes in circulating markers. Cytokines were considered to have a significant change in response to metformin treatment compared to vehicle treatment when fold-change was greater than or equal to ±1.5, according to the manufacturer's criteria. Relative Fold change (FC) is referenced to -vehicle-db/db- based on median. Wilcoxon rank-sum test was used to calculate the -p value-. Threshold for significance: *FC>, or =1.5, or **p<0.05.

Table S4 Pathway enriched by Genego Metcore

Network List

#	Network	GO processes	Total nodes	Seed nodes	Pathways	p-Value	zScore	gScore
1	CXCL13, HGF, MIF, GCP2, Osteoprotegerin	inflammatory response (70.6%; 2.143e-41), chemokine-mediated signaling pathway (35.3%; 8.721e-32), defense response (76.5%; 3.429e-31), positive regulation of response to external stimulus (47.1%; 4.890e-29), cell chemotaxis (41.2%; 2.526e-28)	53	14	1	2.240E-37	102.2	103.45
2	Triacylglycerols intracellular, Cholesteryl ester cytosol, 1-Stearoyl-2-oleoyl lecithin intracellular, Cholesteryl ester lysosome, 1-Palmitoylglycerol 3-phosphoethanolamine (2R) intracellular		50	7	0	3.800E-22	107.46	107.46
3	Triacylglycerols intracellular, Phosphatidylethanolamine intracellular, 1-Acyl-2-linolenoyl-3-sn-phosphatidylcholine (2R) intracellular, Cholesteryl ester endoplasmic reticulum, Cholesteryl linoleate intracellular		50	5	0	3.680E-15	76.74	76.74
4	IL-12 alpha, CCL20, IL-4, MIF, IL-13	positive regulation of macromolecule metabolic process (95.7%; 4.007e-32), response to organic substance (97.8%; 8.187e-32), positive regulation of metabolic process (95.7%; 9.047e-31), regulation of cell proliferation (82.6%; 2.044e-30), positive regulation of cellular metabolic process (93.5%; 3.229e-30)	50	6	0	2.090E-14	45.48	45.48
5	Leptin, APOB, SP1, VLDL, LDL	very-low-density lipoprotein particle assembly (66.7%; 2.203e-16), triglyceride mobilization (66.7%; 2.777e-16), lipoprotein localization (66.7%; 1.138e-15), lipoprotein transport (66.7%; 1.138e-15), plasma lipoprotein particle clearance (66.7%; 3.558e-15)	50	5	0	2.320E-12	43.05	43.05

6	IBP1, IBP2, IGF-1, PI3K cat class IA (p110-alpha), Kallikrein 5	cellular response to organic substance (80.9%; 5.491e-25), response to peptide (51.1%; 2.478e-22), cellular response to chemical stimulus (80.9%; 4.290e-22), response to peptide hormone (48.9%; 1.161e-21), positive regulation of response to stimulus (72.3%; 1.589e-21)	50	4	0	3.210E-09	29.97	29.97
7	Cholesteryl ester extracellular region, Phosphatidylethanolamine extracellular region, Triacylglycerols extracellular region, Cholesterol extracellular region, Oleic acid extracellular region	plasma lipoprotein particle assembly (100.0%; 7.270e-20), plasma lipoprotein particle clearance (100.0%; 7.270e-20), protein-lipid complex assembly (100.0%; 1.208e-19), plasma lipoprotein particle remodeling (100.0%; 1.938e-19), protein-lipid complex remodeling (100.0%; 1.938e-19)	50	3	0	1.870E-07	28.57	28.57
8	G-protein alpha-i family, Pdx-1 (IPF1), CXCR6, CCR9, Neuromedin U receptor 2	positive regulation of cholesterol esterification (36.8%; 2.422e-32), G-protein coupled receptor signaling pathway (89.5%; 4.000e-32), regulation of cholesterol esterification (36.8%; 1.359e-31), regulation of lipase activity (47.4%; 6.438e-30), macromolecular complex remodeling (36.8%; 1.103e-28)	50	3	0	2.970E-07	26.49	26.49
9	Phosphatidyl-1D-myo-inositol intracellular, Phosphatidylinositol intracellular, PtdIns intracellular		3	2	0	3.680E-07	61.41	61.41
10	IL-13, IL-4, IL-6, RelA (p65 NF-kB subunit), Rap1GAP1	regulation of transport (80.9%; 9.108e-28), regulation of localization (85.1%; 4.793e-26), response to insulin (42.6%; 8.209e-23), regulation of phosphorylation (66.0%; 6.564e-22), response to peptide hormone (48.9%; 1.161e-21)	50	3	2	8.130E-07	22.45	24.95
11	IP10, CCL2, Kallikrein 11, G-protein alpha-i2, CCR5	positive regulation of response to stimulus (72.9%; 2.608e-22), positive regulation of cell communication (62.5%; 2.048e-19), positive regulation of signal transduction (60.4%; 2.223e-19), positive regulation of signaling (62.5%; 2.256e-19), inflammatory response (43.8%; 1.455e-18)	50	3	0	8.130E-07	22.45	22.45

12	IBP4, IGF-1, IL-6, IGF-1 receptor, AKT(PKB)	response to peptide (67.3%; 6.558e-36), cellular response to organic substance (91.8%; 1.478e-34), response to peptide hormone (63.3%; 1.497e-33), cellular response to peptide hormone stimulus (55.1%; 3.282e-32), cellular response to peptide (55.1%; 1.805e-31)	52	3	110	8.630E-07	22.22	159.72
